# Age-period-cohort analysis of pancreatitis epidemiological trends from 1990 to 2019 and forecasts for 2044: a systematic analysis from the Global Burden of Disease Study 2019

**DOI:** 10.3389/fpubh.2023.1118888

**Published:** 2023-06-09

**Authors:** Wenkai Jiang, Yan Du, Caifei Xiang, Xin Li, Wence Zhou

**Affiliations:** ^1^The Second Clinical Medical College, Lanzhou University, Lanzhou, China; ^2^The First Clinical Medical College, Lanzhou University, Lanzhou, China; ^3^Department of General Surgery, Lanzhou University Second Hospital, Lanzhou, China

**Keywords:** pancreatitis, Global Burden of Disease, incidence, deaths, age-period-cohort model, prediction

## Abstract

**Objective:**

Pancreatitis poses a serious medical problem worldwide. This study aims to explore the epidemiological trends of pancreatitis from 1990 to 2019, analyze the association between disease burden and age, period and birth cohort, and subsequently present a forecast of pancreatitis incidence and deaths.

**Methods:**

Epidemiologic data were gathered from the Global Health Data Exchange query tool. Joinpoint regression model was used to calculate the average annual percentage changes (AAPCs). Age-period-cohort analysis was utilized to estimate the independent effects of age, period and birth cohort. We also predicted the global epidemiological trends to 2044.

**Results:**

Globally, the incident cases and deaths of pancreatitis increased 1.63-and 1.65-fold from 1990 to 2019, respectively. Joinpoint regression analysis showed that the age-standardized incidence rate (ASIR) and age-standardized death rate (ASDR) decreased over the past three decades. The age effect indicates that older people have higher age-specific incidence and death rates. The period effect on incidence and deaths showed downward trends from 1990 to 2019. The cohort effect demonstrated that incidence and death risk peaked in the earlier birth cohort and were lower in the latest birth cohort. Incident cases and deaths of pancreatitis may significantly increase in the next 25 years. The ASIRs were predicted to slightly increase, while the ASDRs were predicted to decrease.

**Conclusion:**

Epidemiologic patterns and trends of pancreatitis across age, period and birth cohort may provide novel insight into public health. Limitations of alcohol use and prevention strategies for pancreatitis are necessary to reduce future burden.

## 1. Introduction

Pancreatitis is an inflammatory disorder of the pancreas and poses a serious medical problem worldwide (1). When acute pancreatitis develops into severe acute pancreatitis or progresses to chronic pancreatitis, it seriously endangers patient lives and health (2, 3). In the past few years, published studies have shown that the disease burden of pancreatitis significantly affects human health in some places, such as the United States, Europe, China and Australia (4–7). The pancreatitis burden is still increasing owing to the increasing population and risk factors such as alcohol use and cholelithiasis (8).

Studies from some nations have provided epidemiological data on pancreatitis (9, 10). However, in-depth analyses on the effects of age, period and birth cohort remain lacking. Moreover, focusing on the prediction of the incidence and death trends of pancreatitis at the global level is also needed, to facilitate the initiation of more targeted prevention strategies. Therefore, in-depth analysis of pancreatitis epidemiological patterns based on age, period and birth cohort models and predicting future trends may be beneficial for the development of public health and disease prevention.

The Global Burden of Disease (GBD) 2019 study included 369 diseases and injuries in different regions, countries and territories worldwide, which provides an opportunity to perform comprehensive assessments of the epidemiological characteristics of various diseases (11). In this article, we conducted a systematic analysis based on the GBD Study 2019 to explore the epidemiological characteristics of pancreatitis from 1990 to 2019, analyze the age-period-cohort effect on disease burden, and subsequently present a forecast of pancreatitis incidence and deaths, aiming to provide new insight into this digestive system disease.

## 2. Materials and methods

### 2.1. Study design

This study was performed in accordance with the Guidelines for Accurate and Transparent Health Estimates Reporting (12). All detailed information on the disease burden of pancreatitis used in this study can be found in the GBD 2019. All the information about ethical standards is available through the GBD official website.^1^

### 2.2. Data sources

The data were downloaded from the Global Health Data Exchange,^2^ including the annual number of incident case and deaths, and age-standardized rate (ASR) of pancreatitis in GBD regions and all countries/territories, from 1990 to 2019. ASRs can be calculated by summing up the products of the age-specific rates (a_i_, in age group “i”) and the number of population (w_i_, in age group “i”) of the chosen reference standard population, then dividing the sum of standard population weight (13):


$$ASR=\overset{A}{\underset{i=1}{\sum }}a_{i}w_{i}/\overset{A}{\underset{i=1}{\sum }}w_{i}\times 100,000$$

The GBD estimation process is based on identifying multiple relevant data sources, including censuses, household surveys, civil registration and vital statistics, disease registries, health service use, disease notifications and other sources (11). Pancreatitis were mapped to the GBD cause list with the following International Classification of Diseases and Injuries (ICD) codes: K85 (ICD-10) for acute and K86 (ICD-10) for chronic pancreatitis. DisMod-MR 2.1, a Bayesian meta-regression tool, was used to produce estimates by age, sex, year, and location in GBD 2019. The data sources used in estimating the burden of pancreatitis in different locations can be found in the GBD 2019 Data Input Sources Tool.^3^ More information about GBD Study 2019 and data selection steps is described in Supplementary file 1.

We also classified the data by sex, age group and sociodemographic index (SDI). The general methods for the GBD 2019 and the methods for estimations of disease burden have been detailed in previous studies (11, 14). The research results from GBD Risk Factor Collaborators, a comparative risk assessment framework, were used to estimate the proportion of deaths and disability-adjusted life year (DALYs) attributable to the risk factor.

The SDI is a comprehensive indicator of the development status of a country or region. It is based on the overall fertility rate among women under 25 years old, the average education level of individuals aged 15 and older, and the *per capita* income (15). Countries were divided by SDI into five categories (low, low-middle, middle, high-middle, and high).

### 2.3. Joinpoint regression

The Joinpoint regression model, based on the temporal characteristics of the disease distribution, can describe the identification of changes in the recent trend for analyzing disease incidence and death data (16). To determine the magnitude of the time trends for incidence and death rates, the annual percentage change (APC) and average annual percent change (AAPC) were evaluated by Joinpoint regression analysis. The APC was used to estimate the rate of change in a given time period. The AAPC, which provides a summary measure of the APCs over a period of time, was used to assess the trends in the incidence and mortality data of disease for many studies (16).

### 2.4. Age-period-cohort analysis

The statistical modeling of age-period-cohort data often involves the popular multiple classification model, including the effects of age groups, periods of observation and birth cohorts (17). The age effect reflects the impact of changes, including population aging, on morbidity and mortality. The period effect refers to the change in risk of morbidity or mortality at all ages caused by changes in objective factors. Cohort effects are the effects of different levels of exposure to disease risk factors in different birth cohorts on morbidity and mortality. We arranged the pancreatitis incident cases, deaths and global population data into successive five-year periods: from 1990 to 1994 (median 1992) to 2015 to 2019 (median 2017), with 2000 to 2004 (median 2002) as the reference period, and successive age groups with five-year age intervals in all ages (20 groups). Thus, 25 consecutive birth cohorts were also created.

The estimated parameters were obtained from the age-period-cohort web tool,^4^ including net drift, local drift, longitudinal age curves (age-specific rates in reference cohort adjusted for period deviations), period relative risk (ratio of age-specific rates in each period relative to reference period) and cohort relative risk (ratio of age-specific rates in each cohort relative to reference cohort). The methodological details are described in previous literature (18). More details of the age-period-cohort model analysis in this study are shown in Supplementary file 2.

### 2.5. Statistical analysis

Statistical analysis was performed by using R software (Version 4.2.1). Temporal trends of pancreatitis burdens were estimated using the Joinpoint software (Version 4.9.1.0) regression model to calculate APCs and AAPCs. We use relative risk in period and cohort effect to assess the age-specific ratio in each period and each cohort relative to the reference group, respectively. The prediction was conducted in R software through the “Nordpred” package (19). We predicted the number of new cases and ASRs from 2020 to 2044. All rates are reported per 100,000 person-years. The Wald chi-squared test was used to test the significance of the estimated parameters and functions. The “ggplot2” package of R software was used for visualization of all data. A *p* value less than 0.05 was considered to be statistically significant.

## 3. Results

### 3.1. Pancreatitis burden overview

**Figure 1:**
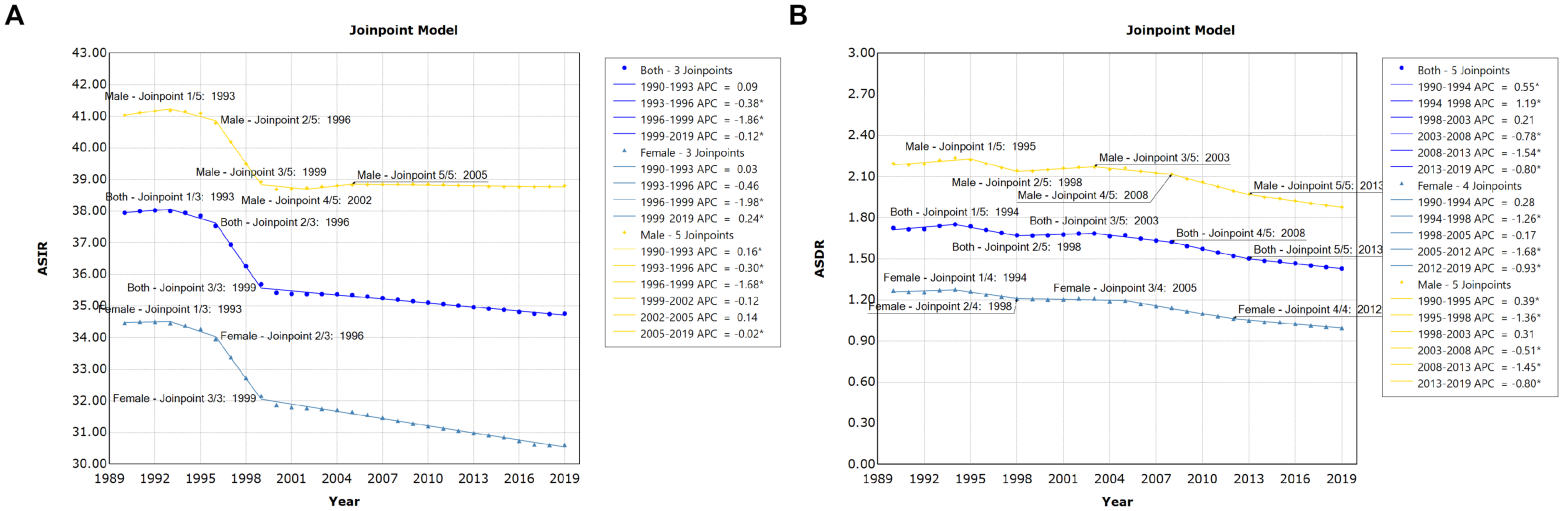
Global trends for ASIR **(A)** and ASDR **(B)** of pancreatitis from 1990 to 2019.

Globally, there were 1,727,789 incident cases of pancreatitis (95% UI, 1,452,132 to 2,059,695 cases) in 1990 and 2,814,972 (95% UI, 2,414,361 to 3,293,591 cases) in 2019, representing an increase of 1.63 times. Pancreatitis caused 115,053 (95% UI, 104,304 to 128,173) deaths globally, including 71,983 (62.6%; 95% UI 63,882 to 81,418) deaths among males in 2019. Joinpoint regression analysis showed that both the age-standardized incidence rate (ASIR) (AAPC = −0.307, 95% CI: −0.357 to −0.257, *p* < 0.001) and age-standardized death rate (ASDR) (AAPC = −0.622, 95% CI −0.770 to −0.473, *p* < 0.001) of pancreatitis presented downward trends from 1990 to 2019. More specifically, the most notable declines in the global ASIR in both males and females were all observed between 1996 and 1999; for the ASDR trend, the most notable declines were observed in 2005 to 2012 for females, and 2008 to 2013 for males, respectively (Figure 1; Supplementary Table S1). The AAPCs in global pancreatitis incidence and death by different age groups from 1990 to 2019 are shown in Table 1. The results demonstrated that ASIR showed upward trends in 5 to 9, 10 to 14, 15 to 19 and 20 to 24 years group; only people in 95 plus age group showed upward trend of ASDR during the past 30 years.

**Table 1:** The average annual percent changes (AAPC) in global pancreatitis incidence and death by age group, 1990 to 2019.

Age group	AAPC and 95% CI (ASIR)	*p* value	AAPC and 95% CI (ASDR)	*p* value
All ages	−0.307 (−0.357 to −0.257)	*p* < 0.001	−0.622 (−0.770 to −0.473)	*p* < 0.001
0 to 4	−0.178 (−0.207 to −0.149)	*p* < 0.001	−1.753 (−1.983 to −1.523)	*p* < 0.001
5 to 9	0.038 (0.013 to 0.063)	*p* = 0.003	−2.176 (−2.371 to −1.981)	*p* < 0.001
10 to 14	0.388 (0.349 to 0.428)	*p* < 0.001	−1.301 (−1.599 to −1.002)	*p* < 0.001
15 to 19	0.524 (0.503 to 0.546)	*p* < 0.001	−0.811 (−1.061 to −0.56)	*p* < 0.001
20 to 24	0.327 (0.281 to 0.373)	*p* < 0.001	−0.517 (−0.742 to −0.291)	*p* < 0.001
25 to 29	−0.029 (−0.068 to 0.011)	*p* = 0.159	−0.32 (−0.554 to −0.085)	*p* = 0.008
30 to 34	−0.203 (−0.23 to −0.176)	*p* < 0.001	−0.594 (−0.755 to −0.432)	*p* < 0.001
35 to 39	−0.269 (−0.286 to −0.251)	*p* < 0.001	−0.417 (−0.76 to −0.074)	*p* = 0.017
40 to 44	−0.352 (−0.39 to −0.313)	*p* < 0.001	−0.556 (−0.733 to −0.378)	*p* < 0.001
45 to 49	−0.38 (−0.422 to −0.338)	*p* < 0.001	−0.682 (−0.916 to −0.447)	*p* < 0.001
50 to 54	−0.512 (−0.57 to −0.453)	*p* < 0.001	−1.118 (−1.332 to −0.903)	*p* < 0.001
55 to 59	−0.391 (−0.469 to −0.312)	*p* < 0.001	−0.655 (−0.943 to −0.366)	*P* < 0.001
60 to 64	−0.478 (−0.58 to −0.375)	*p* < 0.001	−0.816 (−1.032 to −0.6)	*p* < 0.001
65 to 69	−0.45 (−0.501 to −0.398)	*p* < 0.001	−0.772 (−0.972 to −0.571)	*p* < 0.001
70 to 74	−0.465 (−0.515 to −0.414)	*p* < 0.001	−0.778 (−0.939 to −0.616)	*p* < 0.001
75 to 79	−0.542 (−0.579 to −0.506)	*p* < 0.001	−0.766 (−0.941 to −0.591)	*P* < 0.001
80 to 84	−0.518 (−0.56 to −0.477)	*p* < 0.001	−0.506 (−0.66 to −0.352)	*p* < 0.001
85 to 89	−0.63 (−0.691 to −0.568)	*p* < 0.001	−0.302 (−0.503 to −0.1)	*p* = 0.003
90 to 94	−0.636 (−0.71 to −0.561)	*p* < 0.001	−0.053 (−0.157 to 0.052)	*p* = 0.323
95 plus	−0.726 (−0.826 to −0.627)	*p* < 0.001	0.398 (0.215 to 0.581)	*p* < 0.001

AAPC, average annual percentage change; CI, confidence interval; ASIR, age-standardized incidence rate; ASDR, age-standardized death rate.

**Figure 2:**
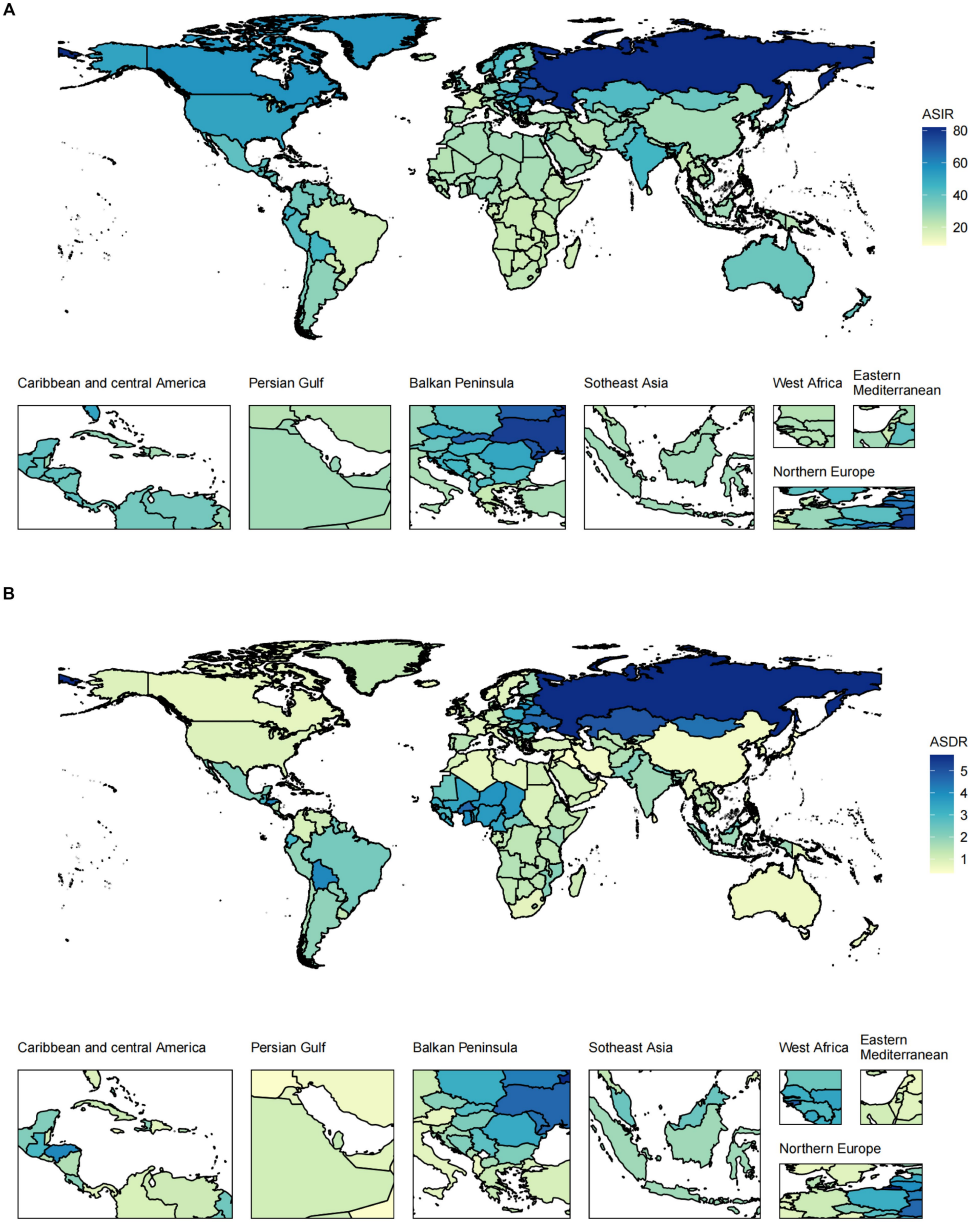
The ASIR **(A)** and ASDR **(B)** of pancreatitis in all countries/territories in 2019.

At the regional level, the highest ASIR of pancreatitis occurred in Eastern Europe in both 1990 and 2019. The region with the highest ASDR changed from Andean Latin America to Eastern Europe (Supplementary Figure S1; Supplementary Tables S2, S3). The ASIR decreased the most in East Asia and increased the most in South Asia. East Europe is the only region where the ASDR showed an upward trend over the past 30 years (Supplementary Figure S2). In all countries/territories, Russia (81.97, 95% UI 70.13 to 95.07) and Ukraine (77.04, 95% UI 65.48 to 90.03) had the highest ASIRs among all countries/territories (Figure 2; Supplementary Table S4). The ASDR followed a very similar pattern: Russia and its neighboring country, Kazakhstan, were the top two countries in 2019 (Supplementary Table S5). In 2019, within the five SDI quintiles, the ASIR of pancreatitis decreased from the high SDI quintile (38.07, 95% UI 33.88 to 42.72) to the low SDI quintile (29.24, 95% UI 24.65 to 34.74), whereas the ASDR was the highest in the low SDI quintile (2.09, 95% UI 1.67 to 2.68) and lowest in the high SDI quintile (0.88, 95% UI 0.81 to 0.99).

### 3.2. Attributable risk

**Figure 3:**
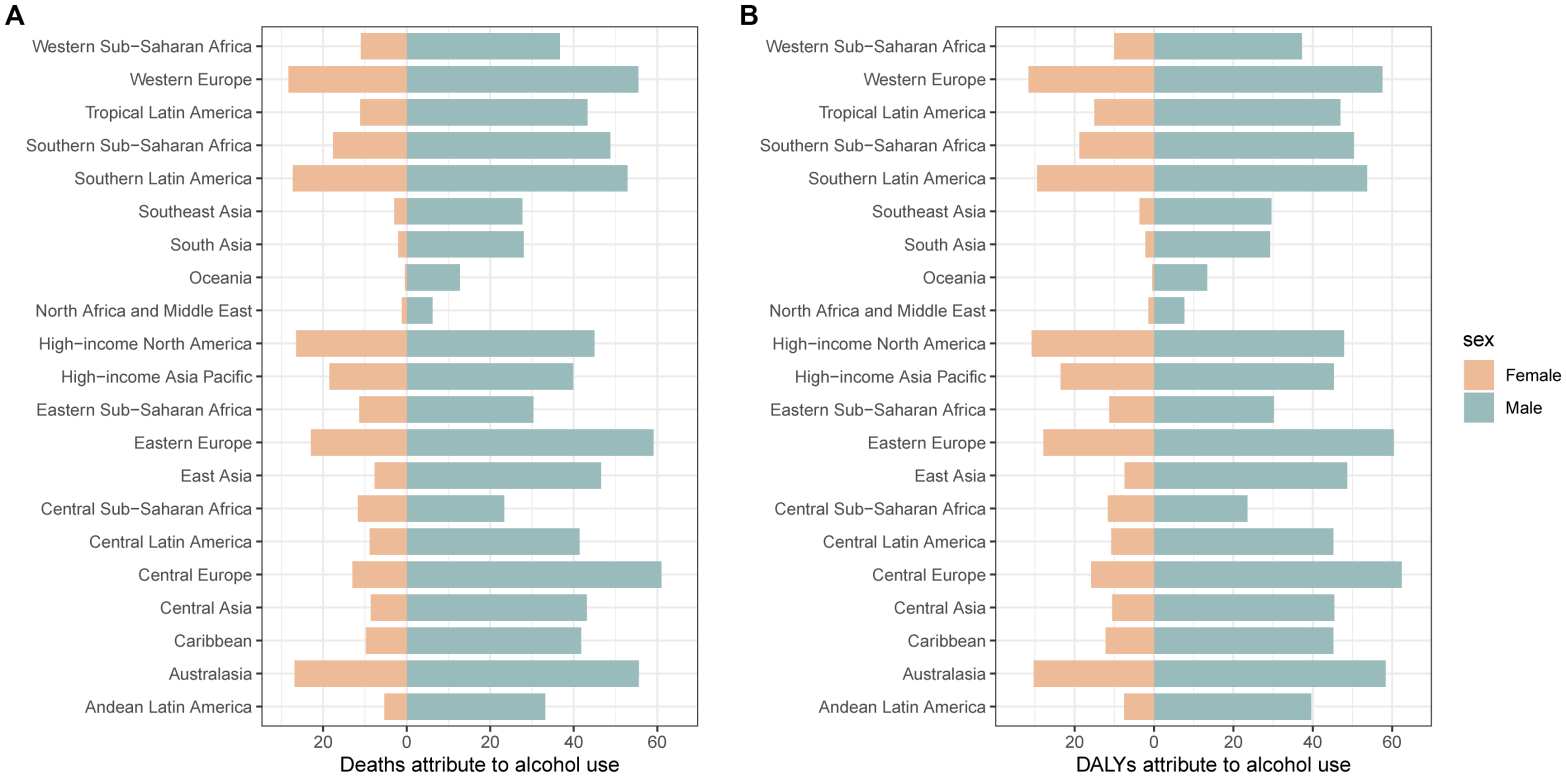
The proportion of deaths and DALYs attributable to alcohol use for pancreatitis in 21 GBD regions for males **(A)** and females **(B)** in 2019. DALYs: disability-adjusted life years.

Alcohol use was the most important risk factor for pancreatitis in people over the age of 15 years. The percent of deaths and DALYs attributable to alcohol use in pancreatitis from 1990 to 2019 are shown in Supplementary Figure S3. In 2019, the proportion of deaths attributable to alcohol use for males was highest in Central Europe; for females, it was highest in Western Europe; the lowest proportions were in Oceania, North Africa and the Middle East, both in males and females (Figure 3). The proportion of pancreatitis attributable to alcohol use also varied by age group and sex. For instance, the proportion of deaths attributable to alcohol use was higher than 40% in males aged between 30 and 69 years. However, alcohol use accounted for approximately 15% among females in the same age group. The proportion of deaths attributable to alcohol increased again when males were older than 85 years old and females were older than 80 years old. The pattern of DALY is similar to that mentioned above (Supplementary Figure S4).

### 3.3. Age-period-cohort model analysis

**Figure 4:**
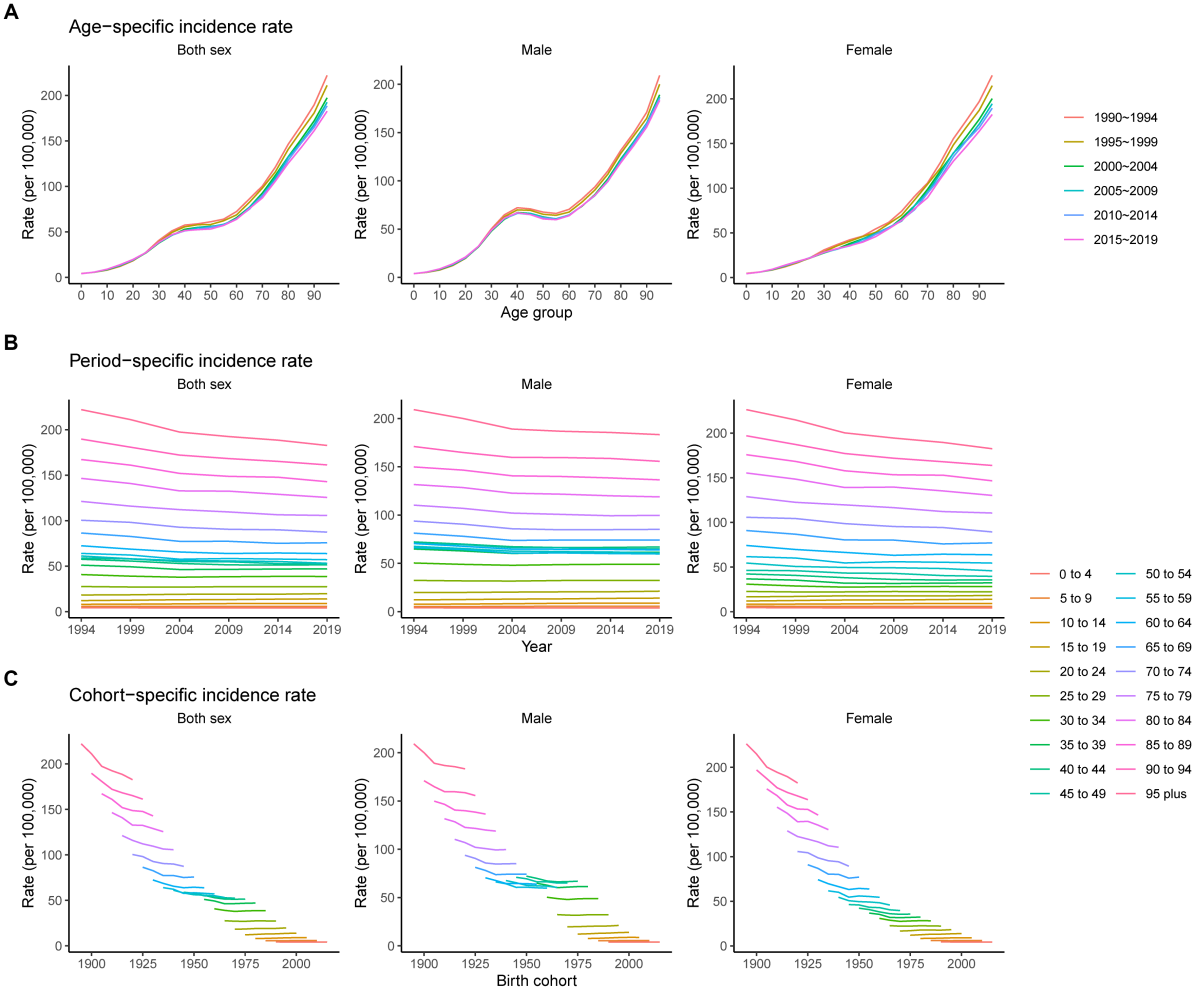
Age-, period-, and cohort-specific incidence rates of pancreatitis from 1990 to 2019. **(A)** Age-specific incidence rate grouped by period; **(B)** period-specific incidence rate grouped by age; **(C)** cohort-specific incidence rate grouped by age.

Trends in the age, period, and cohort-specific incidence rate of pancreatitis are presented in Figure 4. The age-specific incidence rates in males showed a slight downward trend from 40 to 60 years. For females, the rate has always increased with age. The period variations in pancreatitis incidence in both sexes were relatively stable in the younger groups and trended downward over the period in the older age groups (60 to 64, 65 to 69, 70 to 74, 75 to 79, 80 to 84, 85 to 89, 90 to 94, and 95 plus). The cohort-specific rate trends showed that incidence rates for both sexes decreased continuously with advancing year of birth, except for the younger age groups, which showed no significant changes.

**Figure 5:**
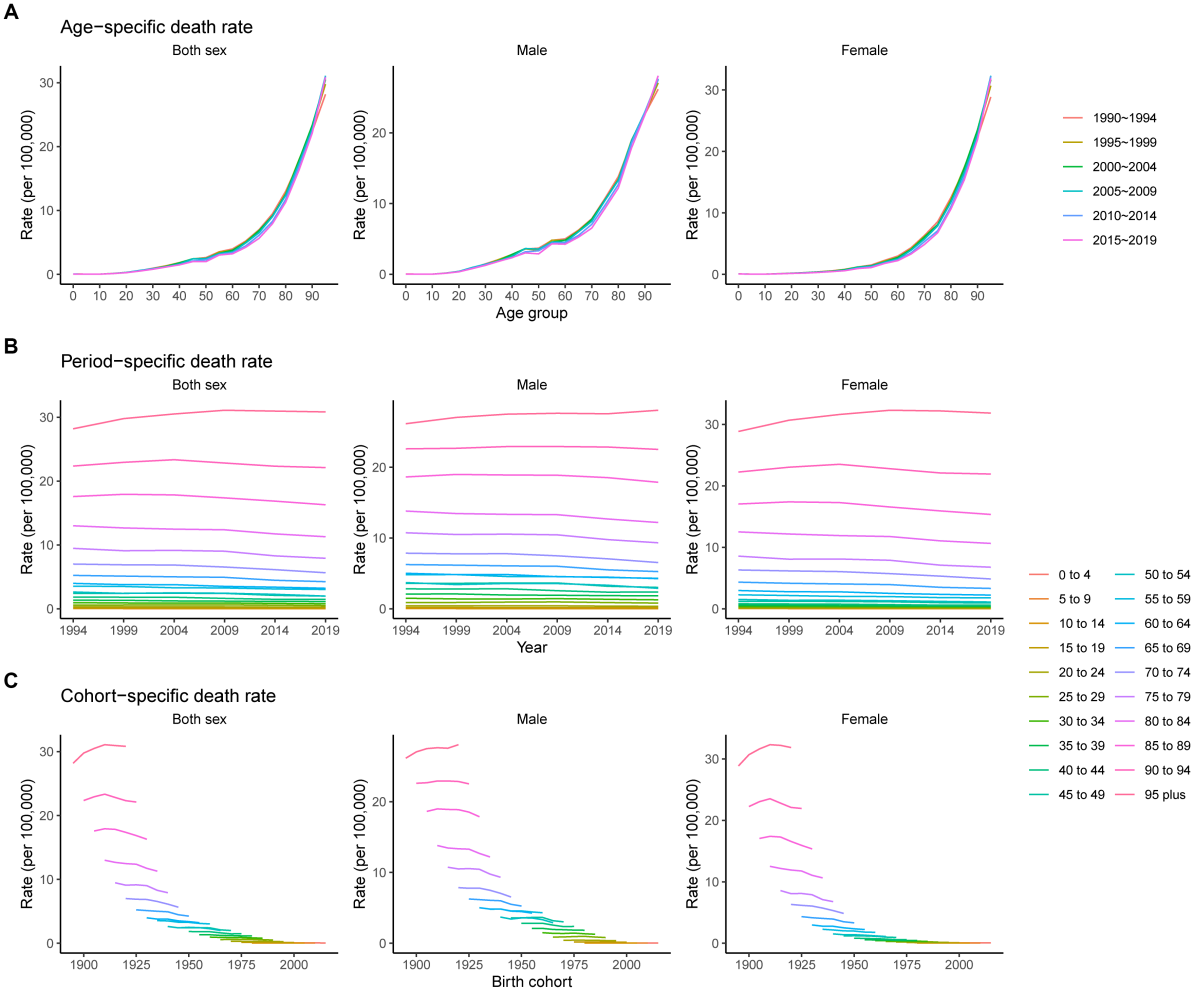
Age-, period-, and cohort-specific death rates of pancreatitis from 1990 to 2019. **(A)** Age-specific death rate grouped by period; **(B)** period-specific death rate grouped by age; **(C)** cohort-specific death rate grouped by age.

Figure 5 shows the age, period, and cohort-specific death rates of pancreatitis. The age-specific death rate accelerated with age, reaching a peak in the oldest age group, and this pattern was consistent across both males and females. Unlike the incidence rate, we noted that some age groups, such as 95 plus, 90 to 94 and 85 to 89 years, showed an increase in the earlier birth cohort and subsequently a decrease in the later birth cohort; other age groups showed continuously downward trends in cohort-specific death rates. The period-specific rate followed the same trend as the cohort-specific rate.

**Figure 6:**
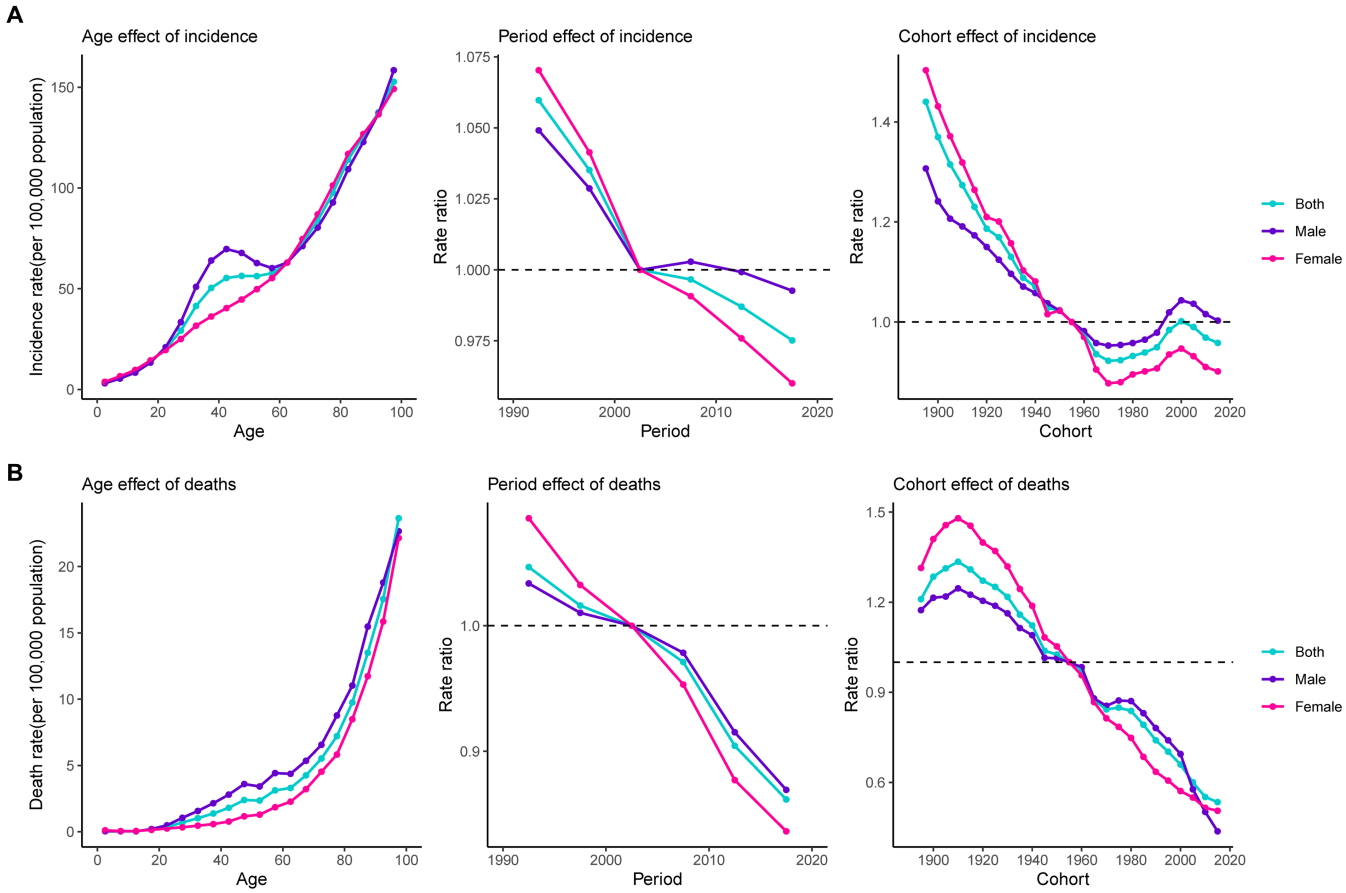
Estimates of age, period, and cohort effects on pancreatitis incidence and death. **(A)** Effect on incidence; **(B)** effect on death.

The global net drift for both sexes demonstrated a decreasing trend in pancreatitis incidence and deaths from 1990 to 2019 (Supplementary Table S6). Supplementary Figure S5 shows the local drifts for both sexes. Age, period, and cohort effects of pancreatitis epidemiological trends for both sexes are presented in Figure 6. Age was significantly associated with pancreatitis incidence and death rate, with the lowest rate in the youngest age group, and with the rate increasing with age. In addition, a sex difference was found in the age effect of incidence between the ages of 25 to 60 years and the age effect of death in people over the age of 20: the rates were higher in males than in females. Period effects generally showed a declining risk of incidence and deaths across the study period and were even more pronounced among females. The cohort effect showed that the incidence risk decreased first; and then incident risk increased first and then declined in the recent birth cohort (1970 to 2000). Compared with people born in the referent 1955 to 1959 cohort, the incidence relative risks and death relative risks decreased for both males and females in the latest birth cohort. In particular, among the recent birth cohorts, males whose birth cohort was from 2000 to 2009 had a higher relative risk than the reference cohort. Age-period-cohort analysis of pancreatitis deaths attributable to alcohol use is shown in Supplementary Figure S6.

### 3.4. Predictions to 2044

**Figure 7:**
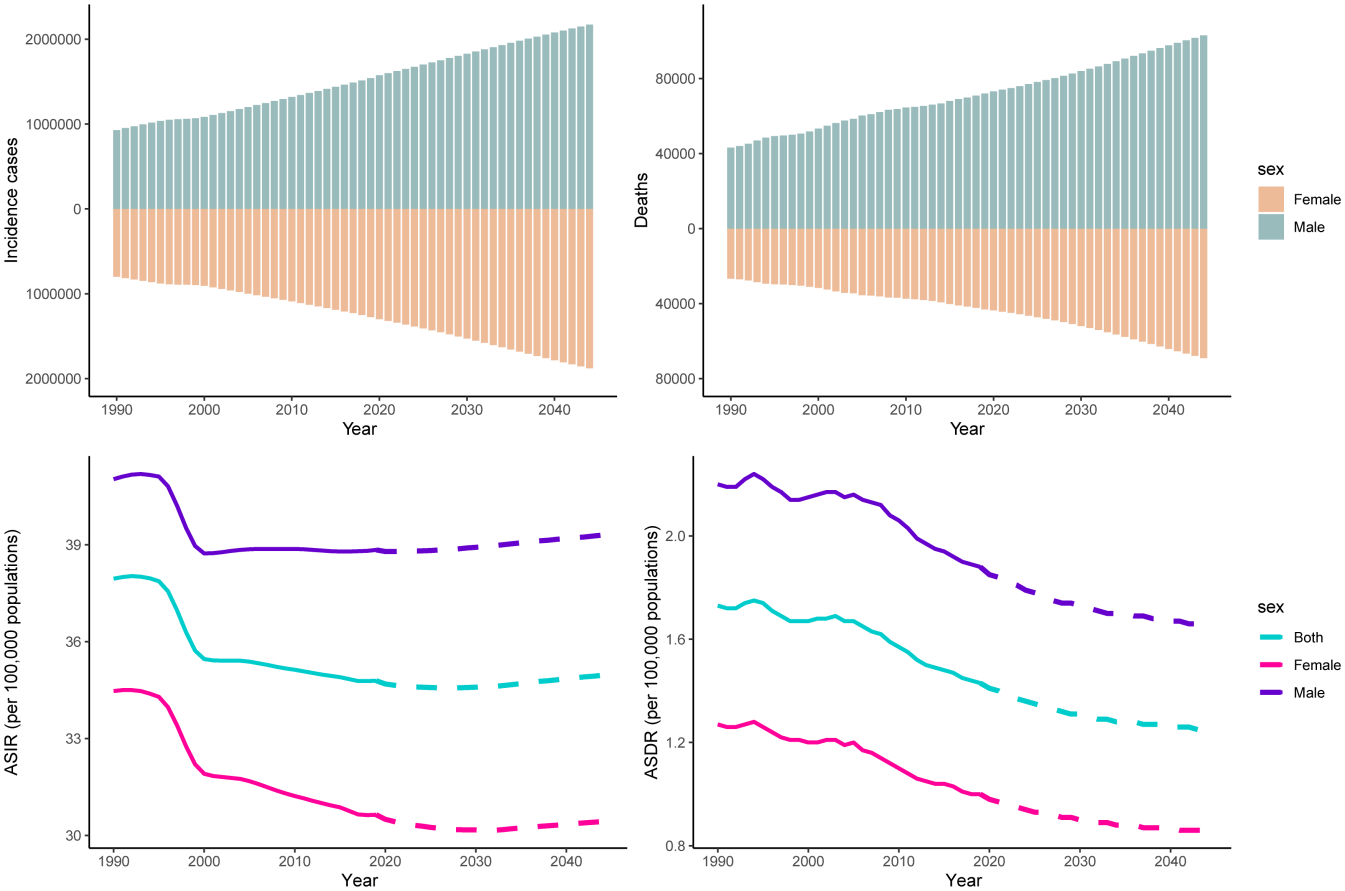
Trends in counts and ASRs of pancreatitis for both sexes worldwide from 1990 to 2044. Note: observed rates are plotted with solid lines, and predicted rates are plotted with dashed lines.

The number of incident cases and deaths from pancreatitis are predicted to continue to increase globally, from 2,814,972 in 2019 to approximately four million in 2044 and from 115,053 in 2019 to approximately 170 thousand in 2044, respectively. Over the next 25 years, the incident cases and deaths number of pancreatitis in males will always be higher than that in females worldwide. Compared to 2019, the ASIR of pancreatitis is expected to rise slightly in 2044 for both sexes. However, as time goes on, the ASDR may show decreasing trends (Figure 7). We also predicted the incidence and death trends of four countries (China, India, Russia and the United States). The results showed that the ASIR for males will slightly increase in India, Russia and the United States; only females in India will show an increase in ASIR (Supplementary Figure S7). For ASDR, only India’s females will show an upward trend (Supplementary Figure S8).

## 4. Discussion

Pancreatitis is a common digestive disorder that is one of the most common causes of gastroenterology-related hospitalizations in the United States (20). At present, the incident cases of pancreatitis continue to rise, causing a huge medical and social burden. Our results showed that the number of incident cases and deaths showed rising trends from 1990 to 2019 and may continue to increase over the next 25 years globally. The large differences in pancreatitis burden between sexes, ages and locations, and the effect of age, period, and birth cohort remind us that the causes should be investigated in depth, such as genetic factors, risk factors, medical technologies and other underlying biological and sociological issues that contribute to human disease.

In 1990 and 2019, the burden of pancreatitis was greater in men than in women. This difference in burden may be related to gender differences in the amount of alcohol consumed by men and women and the degree of adaptation and response to alcohol. Data showed that 76.9% of people who consume harmful amounts of alcohol are male (21). A study of 553,480 adults with acute pancreatitis showed that women had a higher mean age than men (52.81 years versus 50.97 years) (22). Many females are lifelong abstainers. Compared with men, women drink less and are less likely to develop alcohol-related diseases (23). However, women who drink too much have more health problems. Women’s ability to metabolize alcohol and the influence of alcohol on the nervous system and endocrine system may lead to some differences between men and women (23). In addition, women have a higher rate of gallstone pancreatitis, which may be one of the reasons for the higher incidence of pancreatitis in older women (24). However, the relationship between sex differences and pancreatitis at the molecular and genetic levels needs to be further studied.

Trends, patterns, and changes in the burden of pancreatitis vary by region. The current highest burden of pancreatitis is in Eastern Europe, which may be related to the long-term drinking habits of many countries in the region: the history and culture of European countries have a deep and lasting influence on people’s drinking preferences. Globally, Eastern Europe has been repeatedly identified as having the highest levels of alcohol-related health hazards (25). In 2010, WHO member states reached a consensus at the World Health Assembly on a global strategy to tackle the harmful use of alcohol, which mainly includes supply, marketing, price and monitoring (26). In contrast, some North African and Middle Eastern countries prohibit alcohol consumption, thus possibly contributing to the lowest proportion of age-standardized morbidity and mortality in these countries and regions (27).

Due to the intricate interaction among age, period, and cohort factors, we applied the age, period, and cohort model to quantify their net effects on pancreatitis incidence and deaths. It was observed that the age effect increased from the youngest age group to the oldest age group, which is consistent with the trend in several countries (28, 29). The increased disease burden of the older adults may be related to the fact that old people pancreatitis is mostly induced by cholelithiasis or complicated by underlying diseases. Studies have shown that patients over 70 years of age have a higher rate of biliary pancreatitis (30). The incidence of gallstones increases with age. Additionally, advanced age is associated with increased risk of mortality among pancreatitis patients (31). Because the age effect reflects the impact of an aging population; and in the future, the world’s aging degree will increase year by year. Accordingly, it is essential to strengthen the publicity and education of pancreatitis and its risk factors, especially in older adults.

The period effect on pancreatitis death markedly decreased globally, which may be explained by advances in medical care. At present, the treatment of pancreatitis is mainly complete continuous maintenance treatment, including fluid resuscitation, sedation and analgesia and enteral nutrition (32). Over the past few decades, etiology researches, recognition and use of the severity signs, and treatment of complications in pancreatitis have made big progress; improvements in timely and accurate diagnoses, as well as in the care of critically ill patients, contributed to reducing mortality of pancreatitis (8, 33). With the progress of clinical science, such as clinical nutrition, critical care medicine and endoscopic technology, the treatment of pancreatitis has gradually stepped up the direction of standardization. The extensive application of a multi-disciplinary team model and timely management of complications are helpful to improve the prognosis of pancreatitis patients, which is also the inevitable trend of standardized treatment in the future.

The birth cohort effect represents early socioeconomic, behavioral, and environmental factors on the risk of disease. Relative risks of the cohort effect of incidence and deaths initially peaked in the earlier birth cohort and exhibited a downward trend in subsequent decades. This trend was also the result of socioeconomic development and medical advancement all over the world. However, the incidence increased over time in the cohort from 1970 to 2000, mainly because of the bad lifestyle and long-term drinking habits of people born in these cohorts. As the world’s population increases and economies develop, the more recent birth cohort people will be affected by a richer economic life. In addition, we found that among males in the 1995 to 2010 birth cohort, the relative risk of pancreatitis incidence was higher than that in the reference cohort. The increased risk in the recent birth cohort demonstrated that alcohol consumption among adolescents should be given more attention, which is also a potential public health problem worldwide.

Alcohol consumption is one of the most important risk factors for pancreatitis. The prevalence of pancreatitis increased approximately 4-fold in subjects with a history of alcohol abuse compared with those without a history of alcohol abuse (4). Alcohol can affect both the regulatory links of pancreatic neurohormones and the physiological processes of pancreatic acinar cells (34). Alcohol can disrupt intracellular calcium balance through the inositol 1,4,5-triphosphate receptor signaling pathway, leading to increased persistent calcium concentrations in the cytosol (8). The toxicity of high intracellular calcium eventually leads to mitochondrial dysfunction and acinar cell necrosis. Additionally, alcohol can trigger the fusion of lysosomes and zymogen granules, resulting in the accumulation of intracellular zymogen granules and the activation of trypsinogen into trypsin. Chronic pancreatitis has always been associated with alcohol use, and the risk of chronic pancreatitis increases significantly with increasing alcohol consumption (35, 36). After the first onset of acute pancreatitis, the risk of progression to CP is approximately 23% in patients who have reduced but daily alcohol consumption and up to 41% in patients who have the same level of alcohol consumption as before the onset of AP (37). Alcohol metabolites play an important role in the pathogenesis of chronic pancreatitis by inhibiting Erk activity and subsequent direct toxicity to acinar cells through increased oxidative stress (38, 39). However, the relationship between the risk of pancreatitis and the type of alcohol consumed is unclear, and the contribution of beverage type to the risk of pancreatitis needs further study. Thus, rationally allocating medical resources and strengthening the prevention of high-risk groups are also needed.

Predicting the epidemiological trends of pancreatitis is useful for informing the disease burden and assisting in public health and medical resource allocation. In 2044, the incidence and number of deaths are expected to rise. As economic life continues to improve worldwide, people’s living habits will affect the occurrence of many diseases. Causes such as alcohol use and cholelithiasis continue to increase the incidence of pancreatitis. In high-income countries, with better health care and stronger health infrastructure, people tend to receive more comprehensive diagnoses and better treatment. However, in some low-income areas, pancreatitis may be misdiagnosed as other acute abdominal diseases, or the diagnosis is delayed in many cases because of the lack of accurate diagnostic tools; some patients with pancreatitis may not receive comprehensive treatment and may progress to severe pancreatitis. The increasing incidence is also probably because of better diagnostic tools (40). Atlanta Classification in 2012 provides clinicians with more accurate criteria for pancreatitis staging and severity (41). Developments in computed tomography and magnetic resonance imaging, as well as some new clinical scoring systems, improved the diagnosis level of pancreatitis and much easier to determine the severity of pancreatitis (42, 43). As organ failure and infected necrosis increase mortality in necrotizing pancreatitis, early identification of complications, better intensive care management and appropriate interventions will decrease the death rate of pancreatitis (32, 33, 44). Moreover, better health education may be helpful to reduce the disease burden of pancreatitis in the future.

There are several limitations in this study. First, the lack of high-quality and detailed data in some regions and countries, especially in low-income areas, leads to certain information biases in the registry database. As data are absent or inaccuracy in some regions and countries, the burden estimates are dependent on the modeled data instead of typical data from individual level. Thus, we need to be cautious to interpret the data at the national level. Next, due to data limitations, we were unable to further explore the specific types of pancreatitis, such as acute pancreatitis and chronic pancreatitis; also, acute and chronic pancreatitis are two different diseases with some overlap for a small portion of patients. Additionally, owing to the five-year of age group intervals in GBD 2019, our age-period-cohort analysis was performed in periods of 5 years, which might smoothen some slightly variations in age, period and cohort effects. Further work is needed to distinguish between individuals in this study classified as having other risk factors, including cholelithiasis, metabolic disorders and autoimmune diseases. Future research should focus on assessing common risk factors and life patterns that influence pancreatitis incidence, deaths and DALY, especially in countries and regions with large populations or high alcohol consumption. Moreover, differentiation of pancreatitis done by diagnostic criteria are also needed to identify the burden of disease more accurately (41, 45). The economic burden of pancreatitis also deserves attention in future studies.

## 5. Conclusion

Our study provides a comprehensive overview of the global pancreatitis burden from 1990 to 2019 and forecasts it to 2044. The epidemiological trend of pancreatitis varies substantially by sex, region, country/territory and SDI quintile. Age, period, and birth cohort effects also influenced the incidence and death patterns of pancreatitis. Therefore, it is necessary to provide healthy habits education and alcohol use limitation to reduce the pancreatitis disease burden.

## Data availability statement

The original contributions presented in the study are included in the article/supplementary material, further inquiries can be directed to the corresponding author.

## Author contributions

WJ, YD, CX, and WZ designed the study. WJ, YD, and CX drafted the initial manuscript. WJ analyzed the data and performed the statistical analyses. YD performed the visualization. XL and WZ provided the language help. All authors contributed to the article and approved the submitted version.

## Funding

This research was funded by the National Natural Science Foundation of China (82260555); the Science and Technology Projects of Chengguan District in Lanzhou, China (2020-2-11-4); and the Traditional Chinese Medicine Scientific Research Project of Gansu Province, China (GZKP-2020-28).

## Conflict of interest

The authors declare that the research was conducted in the absence of any commercial or financial relationships that could be construed as a potential conflict of interest.

## Publisher’s note

All claims expressed in this article are solely those of the authors and do not necessarily represent those of their affiliated organizations, or those of the publisher, the editors and the reviewers. Any product that may be evaluated in this article, or claim that may be made by its manufacturer, is not guaranteed or endorsed by the publisher.

## Footnotes

^1^http://www.healthdata.org/gbd/2019

^2^https://ghdx.healthdata.org

^3^https://ghdx.healthdata.org/gbd-2019/data-input-sources

^4^https://analysistools.cancer.gov/apc
