# Supplementary material for: Age-period-cohort analysis of pancreatitis epidemiological trends from 1990 to 2019 and forecasts for 2044: a systematic analysis from the Global Burden of Disease Study 2019
Source: Front Public Health. 2023 Jun 9;11:1118888. doi: 10.3389/fpubh.2023.1118888 (PMC10288197; doi:10.3389/fpubh.2023.1118888)
Supplement: Supplementary file 1 [file Data_Sheet_1.pdf]

Supplementary Data

Title: Age-period-cohort analysis of pancreatitis epidemiological trends from 1990 to 2019 and forecasts for 2044: a systematic analysis from the Global Burden of Disease Study 2019

Supplementary file 1: GBD overview

Supplementary file 2: Age-period-cohort model

Supplementary file 3: Supplementary figures

Figure S1 Age-standardized incidence and death rates of pancreatitis in 21 GBD regions by sex, in 2019.

Figure S2 The AAPCs of age-standardized incidence and death rates for pancreatitis in 21 GBD regions. (A) AAPCs of ASIR; (B) AAPCs of ASDR. AAPC: average annual percentage change.

Figure S3 Percentage of pancreatitis (A) deaths and (B) DALYs attributable to alcohol use by sex, 1990 to 2019. DALYs: disability-adjusted life years.

Figure S4 Fraction of pancreatitis age-specific (A) deaths and (B) DALYs attributable to alcohol use by age group for males and females, 2019. DALYs: disability-adjusted life years

Figure S5 Local drift values for global pancreatitis incidence and deaths by sex from 1990 to 2019

Figure S6 Estimates of age, period, and cohort effects on pancreatitis deaths attributable to alcohol use.

Figure S7 Age-standardized incidence rates of pancreatitis from 1990 to 2019, and predicted rates to 2044 in four countries. ASIR: age-standardized incidence rate.

Figure S8 Age-standardized death rates of pancreatitis from 1990 to 2019, and predicted rates to 2044 in four countries. ASDR: age-standardized death rate.

Supplementary file 4: Supplementary tables

Table S1 APCs of global pancreatitis ASIR and ASDR by sex in Joinpoint models, 1990 to 2019

Table S2 The incident cases and ASIR of pancreatitis in 1990 and 2019 among different regions

Table S3 The deaths and ASDR of pancreatitis in 1990 and 2019 among different regions

Table S4 The incident cases and ASIR of pancreatitis in 1990 and 2019 among all countries/territories

Table S5 The deaths and ASDR of pancreatitis in 1990 and 2019 among all countries/territories

Table S6 Net drift for age-period-cohort model of global pancreatitis incidence and deaths

Supplementary file 1 GBD overview

1. The Global Burden of Diseases 2019 (GBD 2019)

GBD 2019 is a collaborative research effort aimed at estimating worldwide population, fertility, morbidity, and mortality. GBD draws on the expertise of an extensive collaborator network from around the world. Incidence data are obtained from individual cancer registries or aggregated databases of cancer registries, such as CI5 (cancer incidence of five continents), SEER, EUERG or NORDCAN. All data contained 369 diseases and injuries in five SDI quintiles, 21 GBD regions and 204 countries/territories.

For pancreatitis, a systematic literature review was conducted to capture studies of prevalence and incidence throughout the world. Pancreatitis is defined as inflammation of the pancreas, including acute pancreatitis and chronic pancreatitis. GBD 2019 developed separate models for acute pancreatitis and chronic pancreatitis. The "acute pancreatitis database" included studies that measured incidence of first episode of acute pancreatitis only, and studies that measured incidence of all acute pancreatitis, including recurrent episodes. Studies were added to the "chronic pancreatitis database" if they employed appropriate International Classification of Diseases and Injuries codes or appropriate clinical, biochemical, and radiographic criteria of chronic pancreatitis. Some studies reported incidence of both acute and chronic pancreatitis were extracted to both databases. Moreover, two databases also included administrative data such as claims data and hospital discharge data from some countries.

2. GBD estimate and measure

The latest version of the data download tool contains core summary results for GBD 2019 (https://vizhub.healthdata.org/gbd-results). The evaluation indicators in GBD 2019 include cause of death or injury, risk factor, etiology, impairment, health-adjusted life expectancy, population, fertility and all-cause mortality. In this

study, we mainly used the two estimates: cause of death or injury and risk factors. The measure of cause of death or injury includes incidence, deaths, and disability-adjusted life years (DALYs). The measure of risk factors includes deaths and DALYs. Metric definitions of measure are listed as follows:

Measure	Number	Rate	Percent in risk factors
Incidence	Number of new cases in the population	New cases per 100,000 population	/
Deaths	Number of deaths in the population	Deaths per 100,000 population	Proportion of deaths for a particular cause relative to deaths from all causes
DALYs	Number of DALYs in the population	DALYs per 100,000 population	Proportion of DALYs for a particular cause relative to DALYs for all causes

Estimates for the GBD 2019 cover the data from 1990 to 2019 for both sexes, male and female. Age information is presented as 5-year age groups. Location contains five SDI quintiles, 21 GBD regions and 204 countries/territories. The SDI is a composite indicator of social development status which strongly correlated with health outcomes, ranging from 0 to 1. It is the geometric mean of total fertility rate in those under 25 years old (TFU25), mean education for those age 15 years or older (EDU15+) and lag-distributed income per capita (LDI). The specific ranges of the three indicators are as follows:

Indicators	Lower Bound	Upper Bound
TFU25	0	3
EDU15+	0 years	17 years
LDI	250 USD	60,000 USD

3. Information classification

The GBD 2019 cause list is organized into a hierarchy (four levels). Level 1 contains

3 groups, named communicable, maternal, neonatal and nutritional diseases, noncommunicable diseases and injuries. The broad group “Digestive diseases”, which includes all diseases in the digestive system, is at Level 2 under the Level 1 group “communicable, maternal, neonatal and nutritional diseases”. GBD 2019 classified all digestive diseases into 10 types according to the International Statistical Classification of Diseases and Related Health Problems, Tenth Revision [ICD-10]. In this study, we selected the disease named “pancreatitis” in Level 3 “Digestive diseases”, which coded K85-K86.9.

4. Data analysis

4.1 age-standardized rate (ASR)

The ASR, which was calculated based on the world standard population, is a measure that can eliminate the influence of population age structure differences to the greatest extent. ASRs were calculated on the basis of the following formula:

$$ASR = \sum_{i=1}^{A} a_i w_i \Big/ \sum_{i=1}^{A} w_i \times 100{,}000$$

The ASR is equal to the sum of the product of the specific age ratio (ai) in age group i and the number (wi) of the selected reference standard population group i divided by the sum of the number of the standard population.

4.2 Joinpoint regression

The Joinpoint software (https://surveillance.cancer.gov/joinpoint/download) has been widely used to study trends in disease morbidity and mortality. The annual percentage change (APC) and the average annual percent change (AAPC) can be calculated in Joinpoint regression model. The APC was used to estimate the rate of change in a given time period; the AAPC was used to assess the trends in the incidence and mortality data over a period of time.

The APC and its 95% confidence interval were calculated based on the following formulas:

$$APC = \left[\frac{y_{x_1} - y_x}{y_x}\right] \times 100 = (e^{\beta 1} - 1) \times 100$$

$$APC_l = 100(e^{\beta_1 - s \times t_d^{-1}(1-0.05/2)} - 1)$$

$$APC_u = 100(e^{\beta_1 + s \times t_d^{-1}(1-0.05/2)} - 1)$$

(β_1: regression coefficient; s: standard error of β_1; d: degree of freedom)

The AAPC and its 95% confidence interval were calculated based on the following formulas:

$$AAPC = (exp\left(\frac{\sum w_i \beta_i}{\Sigma w_i}\right) - 1) \times 100$$

$$AAPC_l = \exp\left\{\ln\left[\left(\left(\frac{AAPC}{100}\right) + 1\right) - Z_{1-\frac{0.05}{2}}\sqrt{\sum \tilde{w}_i^2 \tilde{\sigma}_i^2}\right] - 1\right\}$$

$$AAPC_u = \exp\left\{\ln\left[\left(\left(\frac{AAPC}{100}\right) + 1\right) + Z_{1-\frac{0.05}{2}}\sqrt{\sum \tilde{w}_i^2 \tilde{\sigma}_i^2}\right] - 1\right\}$$

(β_i: regression coefficients corresponding to each interval)

Supplementary file 2 Age-period-cohort model

1. Overview

Age-period-cohort models are the state of art in disease projections, assessing past and recent trends and extrapolating mortality or incidence data into the future. By using age-period-cohort models, we can track disease burden, reveal diversity, provide risk factors, quantify natural history and its evolution, and forecast disease epidemiological trends in the future. The age effect refers to the impact of changes, including population aging, on morbidity and mortality. The period effect refers to the change in risk of morbidity or mortality at all ages caused by changes in objective factors. Cohort effects are the effects of different levels of exposure to disease risk factors in different birth cohorts on morbidity and mortality.

2. Data preparation

Age and period were first divided into 5-year continuous intervals from 0 to 4 years to 95 plus, and from 1990 to 1994 year to 2015 to 2019 year, respectively. Cohort groups are calculated by subtracting the age groups from the period groups. Finally, twenty-five birth cohorts were summarized from 1900 to 1904 cohort, to 2015 to 2019 cohort.

3. Data processing tool

The web tool of age, period, and cohort model was provided by National Cancer Institute database (https://analysistools.cancer.gov/apc).

4. Assessment indicators

In a typical age, period, and cohort model, the age and period intervals must all be equal (five-year age groups should be used with five-year calendar periods). As GBD estimates are produced in five-year age groups with annual data, we arranged all data into a single unit framework by selecting the incident cases, death number and total population counts from the mid-year of six five-year-periods to represent for the specific period.

In the web tool of age, period, and cohort model, we mainly gathered the following estimated parameters: local drift, longitudinal age curve, period rate ratios and cohort rate ratios. The start year are set at 1990, and age at zero. We used the default reference year and cohort: 2000 to 2004 (media: 2002) and 1955 to 1959, respectively. The significance of trends in annual percentage change was tested with a Wald chi-squared test in the web tool.

The explanations of estimated parameters are listed as follows.

Estimated parameters	Interpretation
Local drift	Estimated annual percentage change over time specific to age group
Longitudinal age curve	Fitted longitudinal age-specific rates in reference cohort adjusted for period deviations
Period rate ratios	Ratio of age-specific rates in period p relative to reference period
Cohort rate ratios	Ratio of age-specific rates in cohort c relative to reference cohort

Figure S1 Age-standardized incidence and death rates of pancreatitis in 21 GBD regions by sex, in 2019.

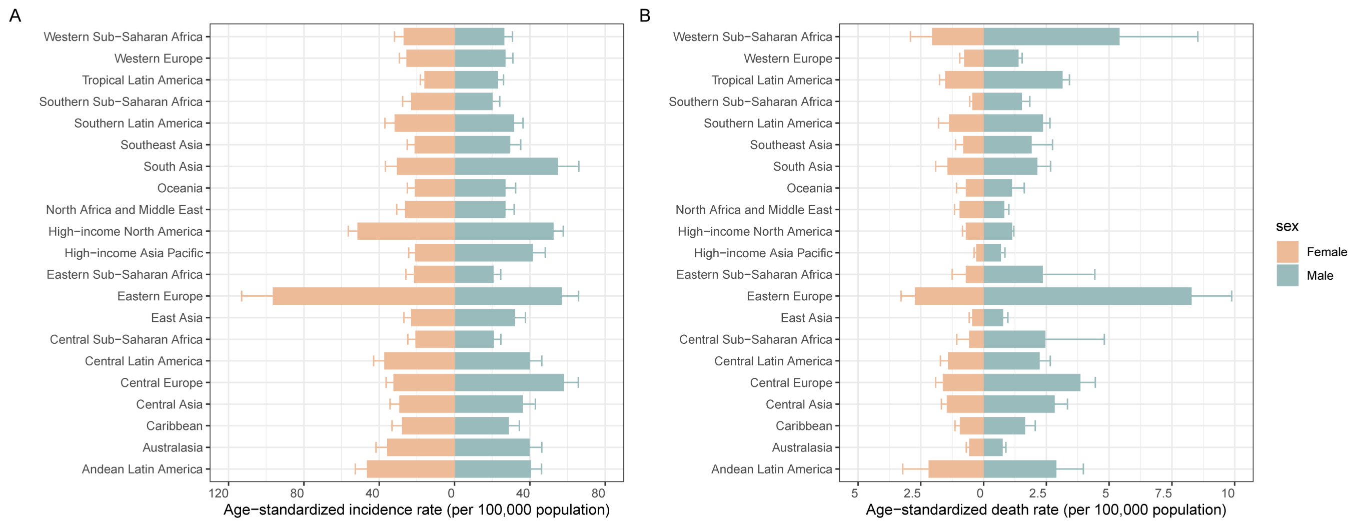

Figure S2 The AAPCs of age-standardized incidence and death rates for pancreatitis in 21 GBD regions. (A) AAPCs of ASIR; (B) AAPCs of ASDR. AAPC: average annual percentage change.

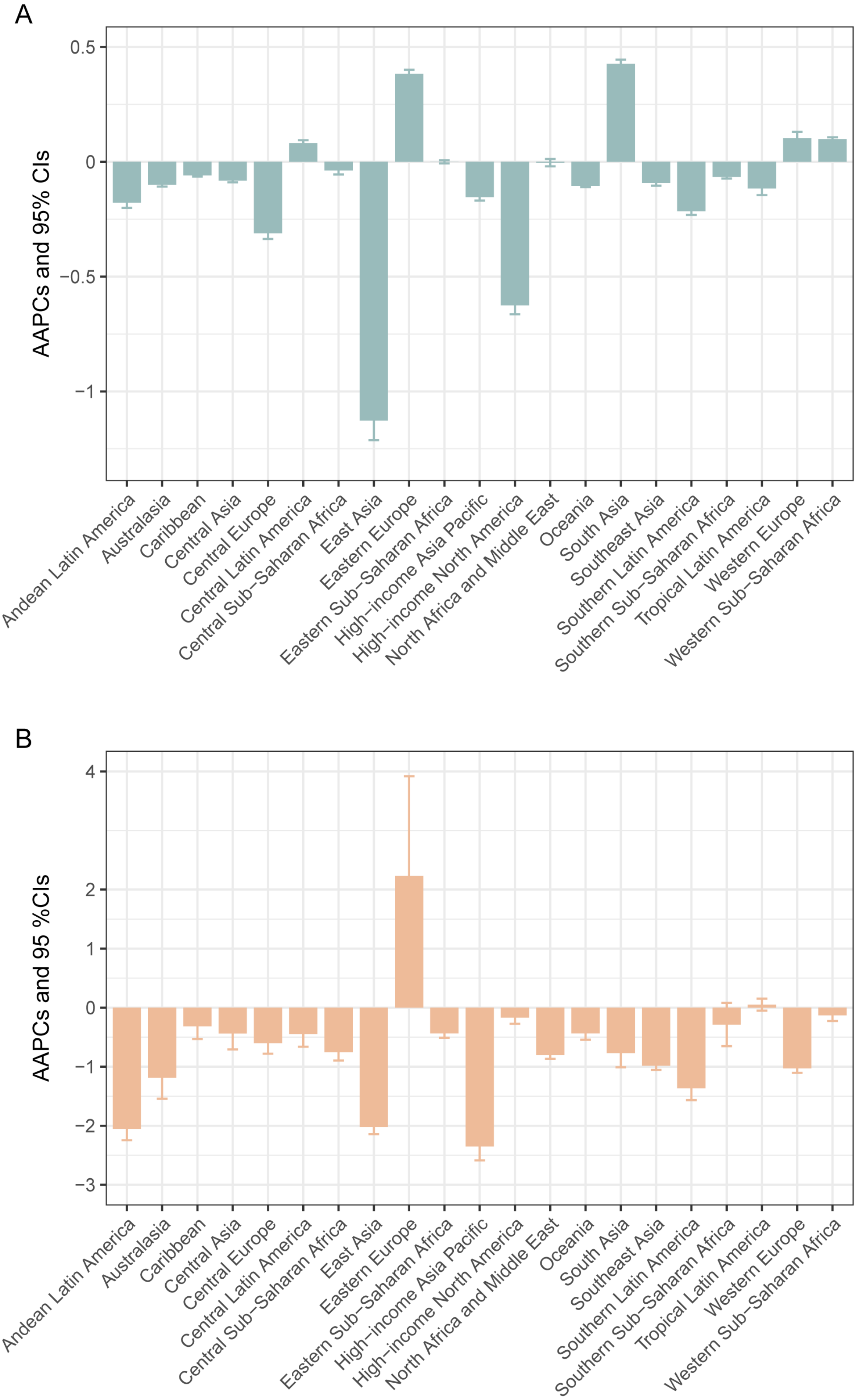

Figure S3 Percentage of pancreatitis (A) deaths and (B) DALYs attributable to alcohol use by sex, 1990 to 2019. DALYs: disability-adjusted life years.

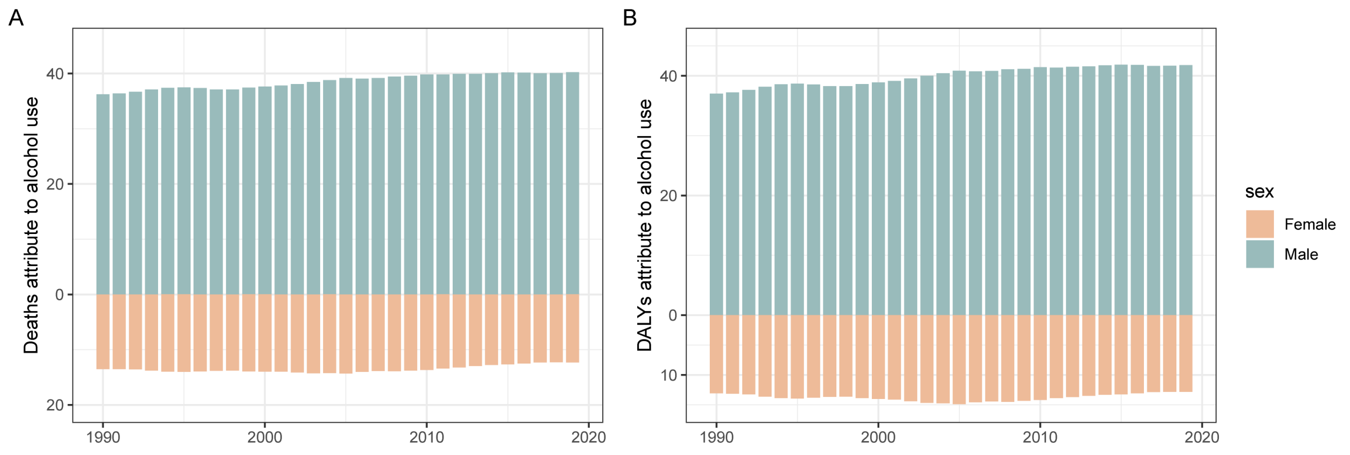

Figure S4 Fraction of pancreatitis age-specific (A) deaths and (B) DALYs attributable to alcohol use by age group for males and females, 2019. DALYs: disability-adjusted life years

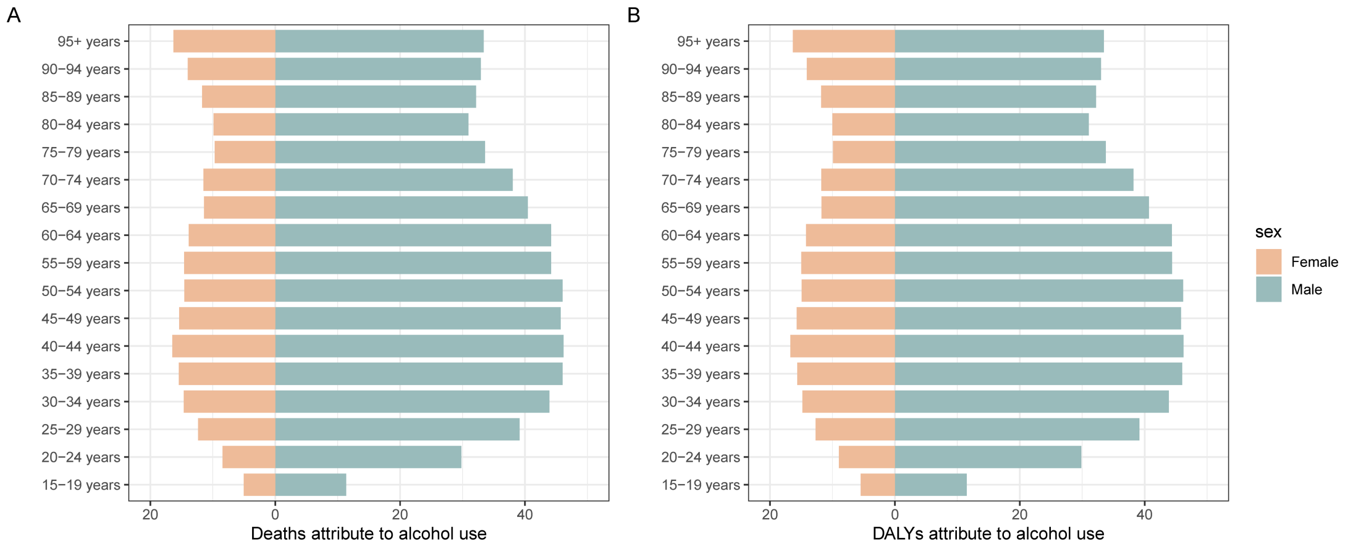

Figure S5 Local drift values for global pancreatitis incidence and deaths by sex from 1990 to 2019

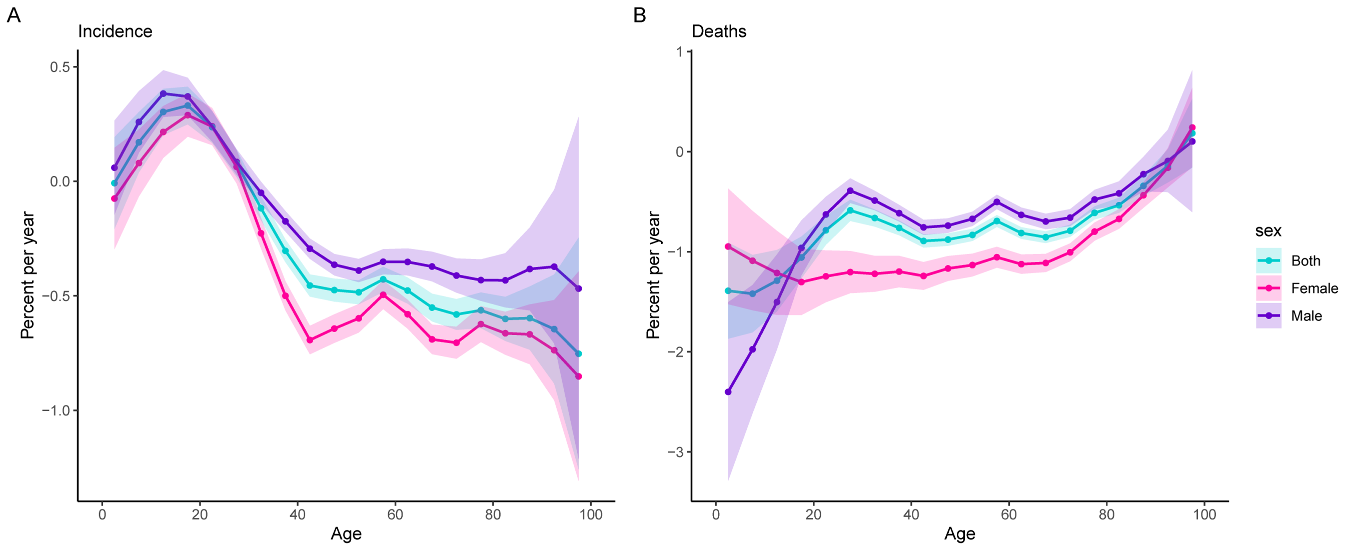

Figure S6 Estimates of age, period, and cohort effects on pancreatitis deaths attributable to alcohol use.

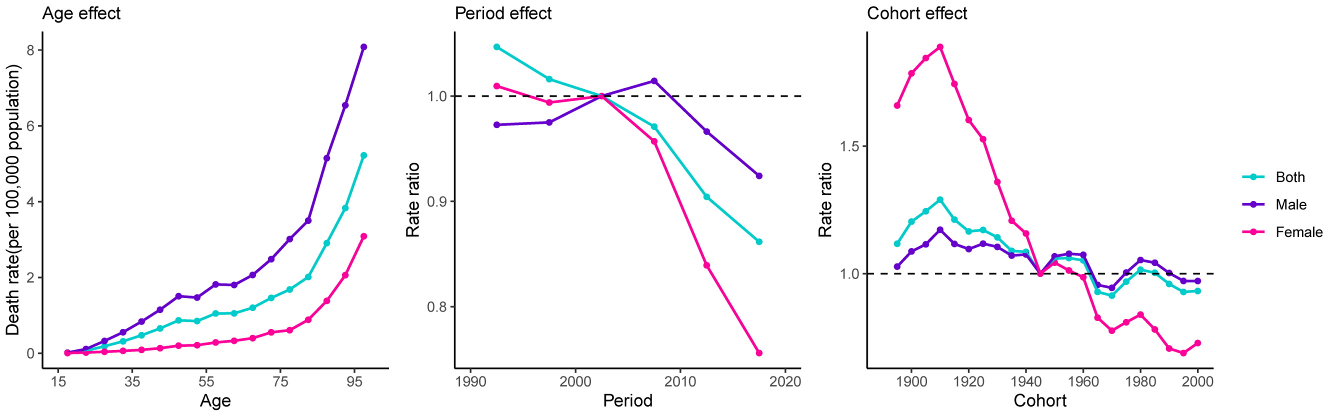

Figure S7 Age-standardized incidence rates of pancreatitis from 1990 to 2019, and predicted rates to 2044 in four countries. ASIR: age-standardized incidence rate.

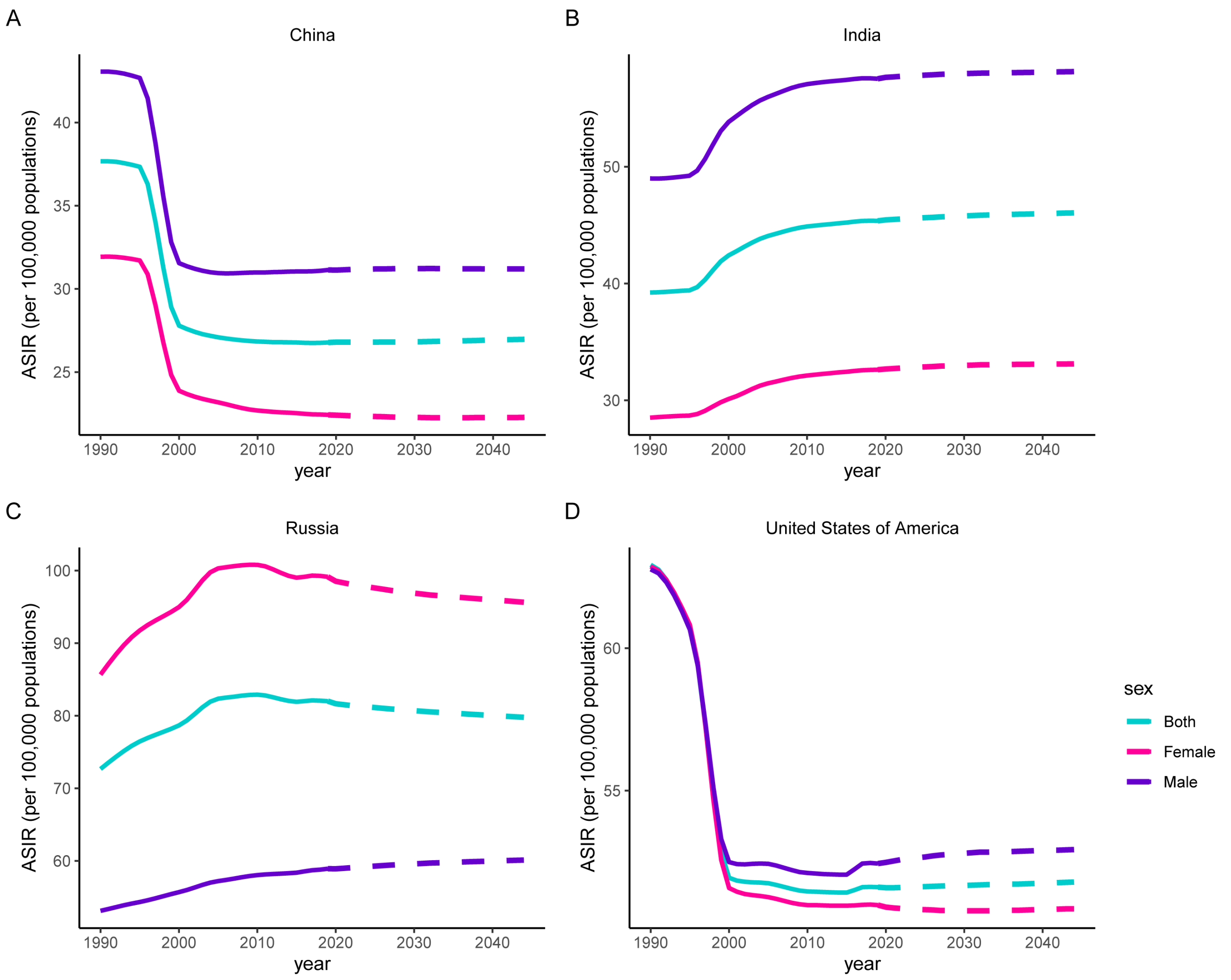

Figure S8 Age-standardized death rates of pancreatitis from 1990 to 2019, and predicted rates to 2044 in four countries. ASDR: age-standardized death rate.

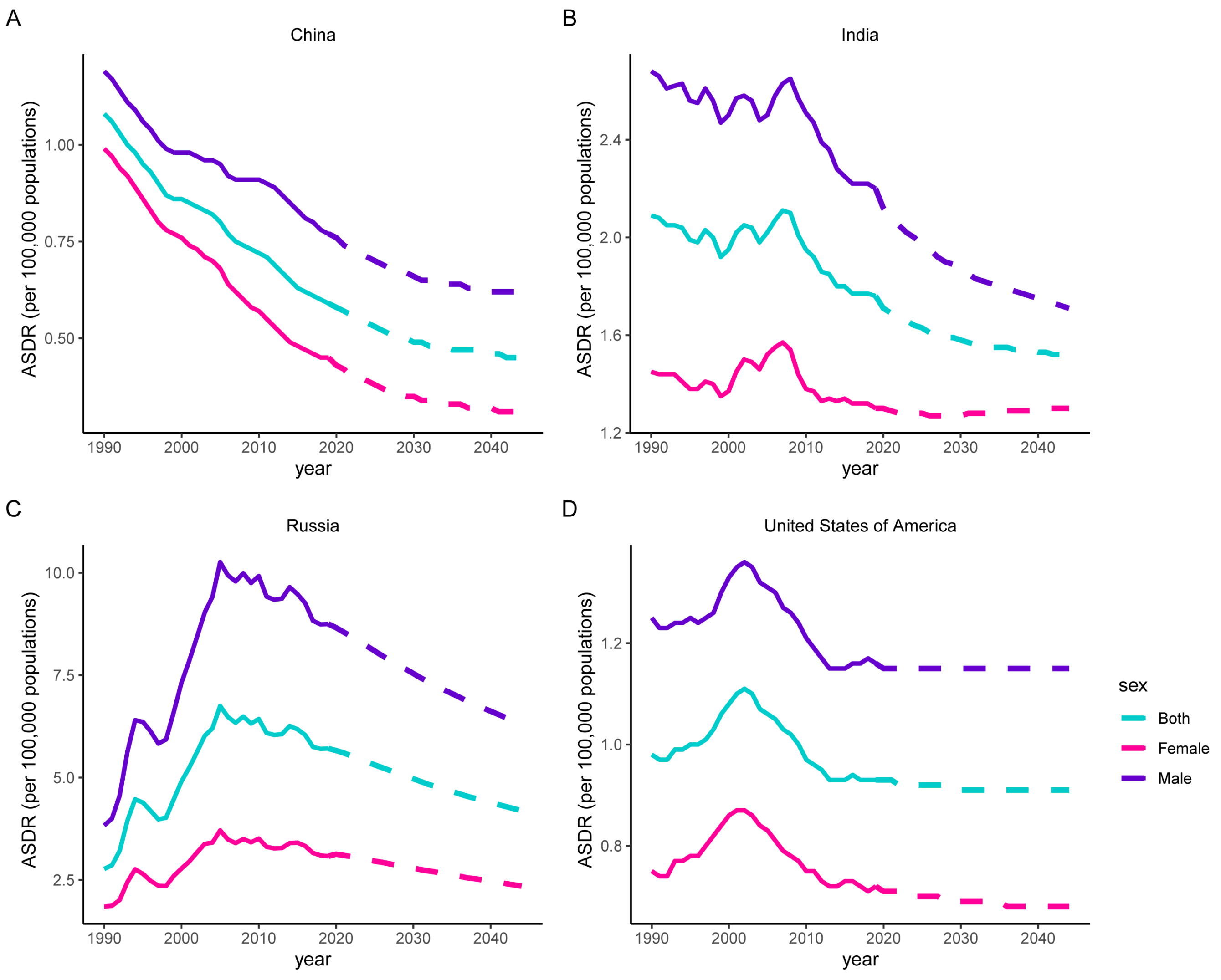

Table S1 APCs of global pancreatitis ASIR and ASDR by sex in Joinpoint models, 1990 to 2019

Segments	ASIR			ASDR		
	Year	APC (95% CI)	P value	Year	APC (95% CI)	P value
**Both**						
Trend 1	1990-1993	0.093 (-0.085 to 0.271)	0.289	1990-1994	0.55 (0.057 to 1.045)	0.031
Trend 2	1993-1996	-0.385 (-0.731 to -0.038)	0.032	1994-1998	-1.187 (-1.882 to -0.487)	0.003
Trend 3	1996-1999	-1.86 (-2.189 to -1.529)	<0.001	1998-2003	0.206 (-0.21 to 0.624)	0.304
Trend 4	1999-2019	-0.12 (-0.129 to -0.111)	<0.001	2003-2008	-0.778 (-1.107 to -0.447)	<0.001
Trend 5	/	/	/	2008-2013	-1.544 (-1.866 to -1.222)	<0.001
Trend 6	/	/	/	2013-2019	-0.805 (-0.99 to -0.619)	<0.001
**Male**						
Trend 1	1990-1993	0.164 (0.081 to 0.247)	0.001	1990-1995	0.389 (0.1 to 0.678)	0.012
Trend 2	1993-1996	-0.303 (-0.464 to -0.141)	0.001	1995-1998	-1.362 (-2.548 to -0.162)	0.029
Trend 3	1996-1999	-1.681 (-1.834 to -1.527)	<0.001	1998-2003	0.312 (-0.048 to 0.673)	0.084
Trend 4	1999-2002	-0.124 (-0.273 to 0.025)	0.096	2003-2008	-0.51 (-0.825 to -0.195)	0.004
Trend 5	2002-2005	0.137 (-0.011 to 0.286)	0.068	2008-2013	-1.448 (-1.744 to -1.151)	<0.001
Trend 6	2005-2019	-0.016 (-0.023 to -0.009)	<0.001	2013-2019	-0.799 (-0.962 to -0.635)	<0.001
**Female**						
Trend 1	1990-1993	0.031 (-0.204 to 0.266)	0.787	1990-1994	0.281 (-0.249 to 0.814)	0.278
Trend 2	1993-1996	-0.462 (-0.921 to 0)	0.05	1994-1998	-1.262 (-2.053 to -0.465)	0.004
Trend 3	1996-1999	-1.979 (-2.405 to -1.551)	<0.001	1998-2005	-0.169 (-0.423 to 0.085)	0.178
Trend 4	1999-2019	-0.242 (-0.253 to -0.23)	<0.001	2005-2012	-1.679 (-1.905 to -1.452)	<0.001
Trend 5	/	/	/	2012-2019	-0.926 (-1.125 to -0.726)	<0.001

APC: annual percentage change; CI: confidence interval; ASIR: age-standardized incident rate; ASDR: age-standardized death rate.

Table S2 The incident cases and ASIR of pancreatitis in 1990 and 2019 among different regions

	Incidence number in 1990	Incidence number in 2019	ASIR in 1990	ASIR in 2019
Global	1727789 (1452132-2059695)	2814972 (2414361-3293592)	37.95 (31.96-44.55)	34.76 (29.8-40.74)
Different SDI				
High SDI	390419 (335677-452157)	533633 (477730-597075)	41.32 (35.36-47.99)	38.07 (33.88-42.72)
High-middle SDI	482857 (406521-568659)	668929 (571034-771708)	43.21 (36.58-50.57)	36.84 (31.63-42.75)
Middle SDI	455055 (373091-554469)	771495 (647793-910393)	33.67 (27.83-40.08)	30.23 (25.57-35.73)
Low-middle SDI	297240 (244737-361676)	602858 (500547-725662)	34.92 (29.04-41.83)	36.78 (30.94-44.04)
Low SDI	101572 (83137-123390)	236892 (194236-286221)	28.41 (23.8-33.69)	29.24 (24.65-34.74)
GBD Region				
Andean Latin America	12752 (10889-14965)	26446 (23242-30102)	45.8 (39.61-52.94)	43.53 (38.36-49.34)
Australasia	8618 (7250-10167)	14395 (12299-16770)	38.65 (32.5-45.26)	37.58 (31.74-44.01)
Caribbean	8808 (7295-10669)	14155 (11773-16693)	28.84 (23.93-34.3)	28.36 (23.55-33.61)
Central Asia	18366 (15467-21700)	29035 (24077-34326)	33.73 (28.41-39.35)	32.93 (27.71-38.49)
Central Europe	67403 (57281-78292)	73016 (64542-82157)	49.4 (42-57.28)	45.17 (40.08-50.95)
Central Latin America	46648 (39437-55592)	96777 (83302-112767)	37.56 (32.12-44.03)	38.55 (33.34-44.74)
Central Sub-Saharan Africa	7427 (6052-9166)	18256 (14929-22374)	21.06 (17.63-25.02)	20.84 (17.45-24.71)
East Asia	396688 (322719-482300)	526066 (444787-615193)	37.96 (31.07-45.39)	27.57 (23.49-32.16)
Eastern Europe	182578 (155057-211692)	221945 (188142-258014)	71.24 (60.79-82.93)	79.59 (68.16-92.54)
Eastern Sub-Saharan Africa	25233 (20531-31140)	57966 (46839-71275)	21.13 (17.66-25.22)	21.14 (17.67-25.22)
High-income Asia Pacific	62652 (52383-74361)	78998 (68905-90261)	32.86 (27.56-39.15)	31.46 (27.29-36.52)
High-income North America	200836 (173238-232437)	257778 (236253-284188)	62.36 (53.74-72)	52.01 (47.5-56.92)
North Africa and Middle East	61824 (50782-75020)	668929 (571034-771708)	26.69 (22.29-31.53)	26.64 (22.51-31.16)
Oceania	1079 (885-1322)	533633 (477730-597075)	24.86 (20.66-29.6)	24.13 (19.96-28.67)
South Asia	328619 (267223-401087)	602858 (500547-725662)	38.09 (31.6-45.89)	42.98 (35.89-51.68)

Southeast Asia	93541 (76473-113689)	236892 (194236-286221)	26.02 (21.63-30.91)	25.33 (21.2-30.08)
Southern Latin America	15849 (13665-18265)	771495 (647793-910393)	33.56 (28.94-38.56)	31.6 (27.36-36.57)
Southern Sub-Saharan Africa	8605 (7042-10531)	140638 (117038-168090)	22.13 (18.46-26.45)	21.71 (18.07-25.94)
Tropical Latin America	23704 (20682-27346)	2386 (1935-2926)	20.06 (17.68-22.74)	19.38 (17.11-21.89)
Western Europe	123271 (107563-140276)	743524 (611176-901906)	25.44 (22.12-29.23)	26.32 (22.81-30.07)
Western Sub-Saharan Africa	33290 (27664-40191)	174246 (143847-208675)	25.98 (22.04-30.59)	26.75 (22.71-31.37)

ASIR: age-standardized incidence rate; SDI: sociodemographic index.

Table S3 The deaths and ASDR of pancreatitis in 1990 and 2019 among different regions

	Deaths in 1990	ASDR in 1990	Deaths 2019	ASDR in 2019
Global	69818 (62047-82529)	1.72 (1.53-2.02)	115053 (104304-128173)	1.43 (1.3-1.59)
Different SDI				
High SDI	12159 (11441-13389)	1.2 (1.13-1.33)	16160 (14601-18476)	0.88 (0.81-0.99)
High-middle SDI	21366 (19824-25093)	2.02 (1.87-2.37)	34393 (31194-37307)	1.79 (1.62-1.94)
Middle SDI	15561 (13214-20288)	1.44 (1.21-1.87)	25776 (22836-30111)	1.09 (0.95-1.26)
Low-middle SDI	14315 (11856-18507)	2.17 (1.76-2.78)	26441 (21686-30711)	1.87 (1.54-2.17)
Low SDI	6386 (4653-8613)	2.41 (1.7-3.29)	12232 (9723-15560)	2.09 (1.67-2.68)
GBD Regions				
Andean Latin America	1077 (811-1299)	4.55 (3.43-5.57)	1445 (1122-1971)	2.54 (1.97-3.48)
Australasia	216 (199-234)	0.95 (0.87-1.02)	339 (292-396)	0.67 (0.58-0.77)
Caribbean	384 (339-437)	1.42 (1.25-1.62)	659 (558-796)	1.29 (1.09-1.56)
Central Asia	1179 (1016-1322)	2.42 (2.06-2.75)	1653 (1382-1884)	2.12 (1.75-2.41)
Central Europe	4477 (4274-5034)	3.2 (3.05-3.62)	5140 (4513-5828)	2.7 (2.37-3.05)
Central Latin America	2018 (1910-2131)	2.06 (1.95-2.21)	4333 (3729-5065)	1.81 (1.55-2.11)
Central Sub-Saharan Africa	452 (302-764)	1.78 (1.15-3.13)	911 (530-1649)	1.43 (0.83-2.68)
East Asia	9324 (7504-12418)	1.08 (0.87-1.45)	11290 (8798-13451)	0.6 (0.47-0.71)
Eastern Europe	7662 (6922-10789)	2.9 (2.62-4.07)	15578 (13367-17735)	5.31 (4.54-6.04)
Eastern Sub-Saharan Africa	1398 (899-2105)	1.69 (1.05-2.6)	2769 (1710-4739)	1.49 (0.91-2.65)
High-income Asia Pacific	1792 (1602-2057)	0.95 (0.85-1.08)	2248 (1887-2804)	0.48 (0.42-0.57)
High-income North America	3348 (3124-3579)	0.97 (0.91-1.04)	5444 (4994-5955)	0.92 (0.85-1)
North Africa and Middle East	1719 (1389-2350)	1.13 (0.87-1.53)	3395 (2655-4069)	0.89 (0.68-1.06)
Oceania	37 (26-52)	1.05 (0.73-1.52)	77 (54-108)	0.93 (0.66-1.31)
South Asia	14050 (11344-19215)	2.25 (1.77-3.08)	25937 (20085-31352)	1.8 (1.39-2.16)
Southeast Asia	4993 (3843-7458)	1.79 (1.38-2.56)	7914 (6541-11171)	1.34 (1.12-1.83)

Southern Latin America	1268 (1136-1374)	2.77 (2.47-3)	1496 (1347-1732)	1.83 (1.65-2.12)
Southern Sub-Saharan Africa	325 (263-415)	1.01 (0.81-1.3)	574 (453-675)	0.93 (0.75-1.08)
Tropical Latin America	2330 (2212-2486)	2.27 (2.13-2.43)	5557 (4794-5987)	2.29 (1.98-2.48)
Western Europe	7987 (7448-8873)	1.45 (1.35-1.59)	9985 (8926-11455)	1.07 (0.98-1.23)
Western Sub-Saharan Africa	3781 (2498-5770)	3.77 (2.51-5.82)	8310 (5927-11883)	3.62 (2.66-5.1)

ASDR: age-standardized death rate; SDI: sociodemographic index.

Table S4 The incident cases and ASIR of pancreatitis in 1990 and 2019 among all countries/territories.

Country/region	Incident cases in 1990	ASIR in 1990	Incident cases in 2019	ASIR in 2019
Afghanistan	2257 (1887-2705)	28.27 (23.77-33.25)	6203 (5045-7565)	28.61 (24.02-33.54)
Albania	1085 (888-1309)	41.11 (34.02-48.67)	1354 (1131-1597)	39.34 (32.78-46.52)
Algeria	4472 (3690-5464)	27.18 (22.7-31.91)	10191 (8377-12187)	26.36 (21.9-31.19)
American Samoa	9 (8-11)	28 (23.36-33.05)	14 (12-17)	28.06 (23.35-33.16)
Andorra	13 (11-16)	22.19 (18.53-26.29)	25 (20-29)	21.04 (17.56-24.9)
Angola	1360 (1107-1674)	20.77 (17.47-24.74)	3959 (3204-4876)	20.63 (17.15-24.47)
Antigua and Barbuda	16 (13-19)	27.8 (22.95-33.28)	27 (22-32)	27.08 (22.53-32.48)
Argentina	11039 (9288-12906)	34.33 (28.91-40.18)	15525 (13149-18264)	30.93 (26.04-36.52)
Armenia	913 (749-1092)	29.7 (24.42-35.23)	1112 (915-1325)	29.98 (24.77-35.45)
Australia	7381 (6149-8734)	39.75 (33.21-46.86)	12151 (10241-14298)	37.52 (31.37-44.44)
Austria	3695 (3417-4000)	38.13 (35.17-41.26)	4985 (4587-5425)	38.24 (35.25-41.65)
Azerbaijan	1699 (1404-2031)	28.56 (23.55-33.86)	3111 (2528-3745)	28.48 (23.46-33.74)
Bahamas	67 (55-83)	31.39 (26.12-37.32)	123 (102-147)	30.4 (25.41-36.08)
Bahrain	113 (89-140)	31.31 (26.28-36.92)	444 (350-543)	29.43 (24.79-34.61)
Bangladesh	23620 (19088-29192)	30.8 (25.26-36.98)	49297 (40635-59626)	32.06 (26.76-38.58)
Barbados	77 (65-93)	28.93 (24.2-34.55)	107 (88-125)	28 (23.31-33.33)
Belarus	7646 (6468-8876)	64.72 (54.86-75.85)	8870 (7517-10296)	69.73 (59.48-81.36)
Belgium	2767 (2345-3221)	22.06 (18.54-25.92)	3325 (2825-3844)	20.87 (17.68-24.45)
Belize	36 (29-43)	27.17 (22.7-32.56)	99 (82-120)	27.52 (22.79-32.75)
Benin	722 (598-874)	24.18 (20.44-28.3)	2006 (1661-2443)	24.69 (21.09-29.01)
Bermuda	21 (17-25)	31.57 (26.13-37.73)	26 (22-31)	29.25 (24.37-35.09)
Bhutan	143 (115-177)	32.73 (27.12-39.46)	248 (203-302)	33.71 (27.93-40.46)
Bolivia (Plurinational State of)	2158 (1797-2594)	48.59 (41.06-57.04)	4627 (3858-5548)	44.6 (37.54-52.65)

Bosnia and Herzegovina	2053 (1704-2437)	45.88 (38.58-54.11)	2059 (1722-2413)	44.95 (37.62-52.82)
Botswana	182 (149-223)	20.98 (17.56-24.98)	403 (331-494)	20.2 (16.88-24.08)
Brazil	23197 (20257-26723)	20.11 (17.73-22.79)	46321 (40840-52388)	19.39 (17.15-21.88)
Brunei Darussalam	47 (38-58)	24.36 (20.49-29.06)	97 (79-118)	22.97 (19.15-27.32)
Bulgaria	4489 (3744-5280)	42.96 (35.84-50.44)	4496 (3758-5336)	43.39 (36.56-51.03)
Burkina Faso	1453 (1214-1764)	24.1 (20.46-28.23)	3752 (3127-4528)	25.57 (21.76-29.79)
Burundi	722 (586-887)	20.24 (16.97-23.96)	1593 (1293-1948)	20.75 (17.31-24.71)
Cabo Verde	60 (50-72)	23.74 (20.02-27.97)	128 (108-152)	24.57 (20.81-29.02)
Cambodia	1642 (1346-2007)	24.49 (20.23-29.24)	3590 (2931-4364)	24.27 (20.03-29.04)
Cameroon	1726 (1442-2067)	25.84 (22.08-30.06)	5125 (4262-6180)	25.79 (22.02-30.05)
Canada	17772 (14934-20955)	57.48 (48.4-67.65)	29038 (24524-34152)	56.19 (47.54-66.06)
Central African Republic	398 (328-487)	22.14 (18.58-26.18)	766 (631-933)	21.56 (18.27-25.48)
Chad	983 (828-1177)	25.66 (21.98-29.91)	2435 (2040-2940)	25.56 (21.9-29.82)
Chile	3721 (3373-4118)	32.21 (29.29-35.64)	7360 (6673-8097)	33.54 (30.45-36.83)
China	380018 (308669-462767)	37.66 (30.77-45.11)	493765 (416705-578675)	26.76 (22.76-31.25)
Colombia	9861 (8096-11967)	38.05 (31.44-45.45)	18314 (15221-21776)	35.81 (29.84-42.68)
Comoros	65 (53-80)	21.17 (17.48-25.25)	125 (103-152)	20.58 (17.16-24.49)
Congo	332 (272-408)	20.82 (17.38-24.77)	812 (658-994)	20.28 (16.94-24.12)
Cook Islands	4 (3-5)	26.73 (22.23-31.72)	6 (5-7)	26.12 (21.65-30.92)
Costa Rica	1046 (862-1263)	42.74 (35.72-50.75)	2067 (1723-2459)	40.64 (33.93-48.47)
Croatia	2861 (2581-3168)	48.21 (43.67-53.35)	3012 (2763-3287)	45.59 (41.89-49.7)
Cuba	3079 (2545-3714)	28.48 (23.5-34.1)	4303 (3605-5046)	29.2 (24.31-34.62)
Cyprus	175 (149-205)	22.14 (19.01-25.75)	379 (331-430)	21.61 (18.99-24.44)
Czechia	6353 (5367-7435)	52.56 (44.49-61.51)	7695 (6819-8712)	50.38 (44.6-57.13)
Côte d'Ivoire	1815 (1496-2204)	24.34 (20.81-28.49)	4524 (3754-5451)	24.74 (21.1-28.86)
Democratic People's Republic of	7223 (5906-8678)	39.8 (33.15-47.2)	12274 (10003-14622)	39.82 (33.03-47.32)

Korea				
Democratic Republic of the Congo	5129 (4174-6346)	21.06 (17.59-25.03)	12235 (10022-14970)	20.93 (17.55-24.74)
Denmark	1413 (1232-1615)	21.65 (18.72-24.83)	1738 (1517-1999)	21.6 (18.5-24.92)
Djibouti	59 (47-74)	20.11 (16.7-24.09)	185 (149-228)	19.44 (16.06-23.18)
Dominica	20 (17-24)	29.82 (24.92-35.44)	23 (19-27)	29.05 (24.17-34.6)
Dominican Republic	1445 (1178-1769)	26.79 (22.14-32)	2761 (2276-3325)	26.56 (21.99-31.71)
Ecuador	3130 (2850-3455)	43.42 (39.89-47.71)	7754 (7416-8093)	47.05 (44.98-49.16)
Egypt	10582 (8703-12780)	27.16 (22.61-32.1)	21875 (17963-26391)	27.46 (22.98-32.44)
El Salvador	1598 (1324-1923)	40.09 (33.73-47.49)	2263 (1894-2715)	36.86 (30.91-44.22)
Equatorial Guinea	61 (50-75)	22.01 (18.44-26.05)	198 (160-245)	21.22 (17.68-25.03)
Eritrea	371 (301-460)	20.35 (16.93-24.27)	949 (766-1171)	20.48 (17.16-24.47)
Estonia	1159 (977-1351)	63.42 (53.58-74.12)	1178 (991-1387)	62.85 (53.01-73.74)
Eswatini	101 (82-124)	20.39 (16.94-24.22)	175 (142-216)	20.23 (16.83-24.02)
Ethiopia	7236 (5842-8924)	22.37 (18.57-26.74)	15756 (12703-19479)	21.9 (18.2-26.21)
Fiji	135 (110-166)	24.79 (20.54-29.55)	201 (163-242)	23.89 (19.67-28.42)
Finland	1984 (1796-2183)	31.69 (28.75-34.8)	2725 (2468-3029)	33.49 (30.24-37.06)
France	14830 (12524-17237)	21.2 (17.81-24.91)	18571 (15681-21737)	19.45 (16.3-23.13)
Gabon	148 (122-182)	20.73 (17.25-24.75)	286 (233-349)	20.05 (16.72-24)
Gambia	156 (129-189)	25.73 (21.97-30.05)	387 (322-467)	25.2 (21.49-29.45)
Georgia	1604 (1315-1907)	27.13 (22.23-32.38)	1287 (1172-1425)	26.83 (24.25-29.74)
Germany	24980 (20867-29136)	24.11 (20.06-28.56)	37431 (31650-43580)	29.13 (24.46-33.94)
Ghana	2418 (2011-2927)	24.6 (20.94-29.06)	6042 (5022-7223)	25.01 (21.18-29.16)
Greece	2754 (2329-3212)	21.57 (18.2-25.33)	3483 (2942-4054)	20.96 (17.46-24.75)
Greenland	28 (23-33)	57.46 (48.49-67.47)	36 (30-43)	55.46 (46.56-65.03)
Grenada	21 (17-25)	28.04 (23.15-33.53)	29 (24-35)	26.32 (22-31.39)

Guam	28 (23-34)	25.6 (21.06-30.47)	44 (37-53)	24.9 (20.52-29.84)
Guatemala	2398 (1984-2886)	43.32 (36.36-51.14)	5915 (4930-7094)	39.66 (33.52-47.02)
Guinea	1019 (852-1218)	23.92 (20.22-27.96)	1974 (1634-2391)	23.72 (20-27.82)
Guinea-Bissau	165 (138-199)	26.43 (22.72-30.74)	321 (267-387)	25.44 (21.82-29.65)
Guyana	175 (142-213)	30.31 (25.26-35.96)	215 (179-259)	29.92 (25.07-35.51)
Haiti	1353 (1107-1636)	29.69 (24.77-34.96)	2925 (2403-3526)	29.3 (24.43-34.7)
Honduras	1316 (1083-1595)	41.16 (34.76-48.74)	3274 (2728-3959)	40.2 (33.86-47.73)
Hungary	6351 (5390-7347)	50.77 (43.07-59.1)	6337 (5320-7450)	45.02 (37.92-52.73)
Iceland	54 (45-64)	20.02 (16.54-23.88)	98 (83-116)	22.29 (18.8-26.29)
India	271027 (220901-330346)	39.27 (32.59-47.29)	618862 (508187-749921)	45.22 (37.71-54.41)
Indonesia	40776 (33346-49429)	28.62 (23.8-33.89)	72002 (58941-86950)	27.77 (23.1-33.09)
Iran (Islamic Republic of)	8894 (7278-10853)	24.42 (20.39-28.85)	19555 (16084-23289)	23.87 (19.99-28.21)
Iraq	2805 (2306-3441)	25.51 (21.06-30.26)	7591 (6223-9257)	23.8 (19.75-28.19)
Ireland	719 (603-852)	19.31 (15.99-22.91)	1160 (962-1367)	18.89 (15.68-22.31)
Israel	958 (800-1143)	20.26 (16.86-23.99)	2003 (1672-2354)	19.77 (16.37-23.35)
Italy	20579 (17815-23781)	27.14 (23.63-31.02)	27615 (24442-31442)	25.57 (22.86-28.73)
Jamaica	536 (445-649)	26.93 (22.37-32.31)	781 (646-941)	26.08 (21.6-31.42)
Japan	51755 (43089-61409)	35.45 (29.62-42.47)	60693 (53258-68867)	36.17 (31.55-41.82)
Jordan	878 (712-1067)	38.49 (32.35-45.35)	3807 (3195-4543)	40.44 (34.29-47.27)
Kazakhstan	6413 (5492-7410)	44.75 (38.54-51.24)	8054 (6859-9290)	42.95 (36.94-49.4)
Kenya	3378 (2726-4171)	23.74 (19.83-28.29)	8680 (7041-10651)	23.89 (19.98-28.48)
Kiribati	15 (12-18)	28.22 (23.64-33.17)	25 (21-31)	27.96 (23.46-32.84)
Kuwait	365 (287-453)	28.63 (23.73-34)	1206 (949-1475)	28.2 (23.41-33.32)
Kyrgyzstan	1097 (908-1302)	31.62 (26.14-37.57)	1835 (1506-2185)	32.18 (26.64-38.1)
Lao People's Democratic Republic	694 (572-841)	24.32 (20.23-28.97)	1465 (1197-1776)	24.21 (20.07-28.74)

Latvia	1982 (1731-2272)	63.57 (55.43-73.42)	1655 (1472-1848)	61.67 (54.58-69.45)
Lebanon	713 (595-857)	27.5 (23.01-32.54)	1423 (1174-1689)	26.9 (22.33-31.87)
Lesotho	271 (224-331)	21.07 (17.64-24.91)	364 (299-444)	21.14 (17.71-25.19)
Liberia	321 (268-387)	23.19 (19.58-27.23)	812 (670-986)	23.55 (19.88-27.72)
Libya	752 (615-919)	28.02 (23.09-33.1)	1771 (1428-2129)	27.44 (22.69-32.45)
Lithuania	2477 (2215-2772)	59.58 (53.26-66.83)	2648 (2372-2950)	64.83 (57.78-72.53)
Luxembourg	126 (110-142)	26.52 (23.23-30.08)	242 (218-268)	29.18 (26.23-32.28)
Madagascar	1467 (1187-1819)	18.97 (15.74-22.82)	3564 (2887-4385)	19.47 (16.23-23.38)
Malawi	1198 (973-1474)	19.9 (16.49-23.57)	2494 (2021-3084)	20.64 (17.22-24.51)
Malaysia	3641 (2996-4373)	27.19 (22.89-32.22)	8262 (6863-9876)	26.76 (22.45-31.57)
Maldives	28 (23-35)	20.53 (16.79-24.85)	104 (81-129)	20.16 (16.51-24.26)
Mali	1342 (1115-1614)	23.58 (19.9-27.69)	3446 (2852-4144)	25.32 (21.59-29.61)
Malta	71 (60-83)	17.25 (14.67-20.16)	123 (108-142)	17.95 (15.68-20.4)
Marshall Islands	7 (6-9)	27.23 (22.68-32.17)	12 (10-15)	26.64 (22.11-31.49)
Mauritania	341 (284-411)	24.54 (20.75-28.78)	702 (586-847)	24.04 (20.24-28.27)
Mauritius	263 (218-314)	26.65 (22.45-31.37)	377 (314-449)	24.39 (20.35-29.05)
Mexico	23224 (20069-27130)	36.46 (31.8-41.85)	50891 (44796-57816)	40.35 (35.63-45.58)
Micronesia (Federated States of)	18 (15-22)	27.66 (23.06-32.68)	22 (18-27)	26.51 (22.21-31.41)
Monaco	10 (8-11)	20.01 (16.65-23.76)	12 (10-14)	19.54 (16.28-23.2)
Mongolia	545 (464-649)	39.59 (33.6-46.12)	1194 (991-1413)	38.64 (32.9-44.83)
Montenegro	281 (234-330)	44.25 (37-51.91)	336 (281-396)	42.35 (35.3-49.98)
Morocco	4883 (4025-5902)	27.31 (22.79-32.13)	9292 (7657-11080)	27.05 (22.53-31.98)
Mozambique	1799 (1479-2210)	20.77 (17.39-24.62)	3889 (3172-4795)	21.13 (17.74-25.06)
Myanmar	7055 (5759-8648)	22.7 (18.8-27.15)	11497 (9408-13908)	21.64 (17.87-25.95)
Namibia	206 (168-253)	20.98 (17.52-24.99)	405 (335-492)	21.11 (17.57-25.05)
Nauru	2 (1-2)	26.23 (21.81-31.13)	2 (2-2)	25.75 (21.48-30.53)

Nepal	3746 (3075-4570)	26.41 (22.02-31.51)	6949 (6074-7975)	25.86 (22.7-29.44)
Netherlands	1667 (1383-1954)	9.17 (7.59-10.78)	2259 (1882-2669)	8.76 (7.17-10.35)
New Zealand	1237 (1084-1413)	33.1 (28.94-37.64)	2244 (2009-2517)	37.86 (33.95-42.35)
Nicaragua	993 (811-1218)	38.35 (32.2-46)	2089 (1710-2529)	36.01 (29.85-43.07)
Niger	1205 (1000-1457)	25.45 (21.71-29.66)	3442 (2859-4169)	26.19 (22.52-30.43)
Nigeria	17216 (14332-20806)	27.45 (23.32-32.42)	41313 (34127-49831)	28.54 (24.25-33.56)
Niue	1 (0-1)	26.43 (21.85-31.46)	0 (0-1)	26.11 (21.7-31.02)
North Macedonia	858 (711-1019)	43.4 (36.33-51.25)	1162 (972-1376)	42.32 (35.77-49.9)
Northern Mariana Islands	9 (7-12)	26.45 (21.93-31.35)	13 (10-16)	26.51 (21.99-31.5)
Norway	1599 (1350-1867)	29.94 (25.23-35.09)	2857 (2480-3284)	40.32 (34.97-46.47)
Oman	314 (251-392)	25.49 (21.11-30.34)	931 (723-1169)	24.65 (20.42-29.32)
Pakistan	30083 (24671-36530)	37.12 (30.92-44.53)	68167 (55967-82921)	39.02 (32.71-46.71)
Palau	3 (3-4)	26.28 (21.95-31.15)	6 (4-7)	26.41 (21.8-31.4)
Palestine	358 (294-437)	29.18 (24.42-34.51)	983 (805-1187)	28.2 (23.54-33.28)
Panama	720 (596-877)	36.29 (30.12-43.4)	1470 (1211-1757)	35.03 (28.95-41.91)
Papua New Guinea	657 (538-807)	24.27 (20.12-28.98)	1680 (1356-2061)	23.7 (19.56-28.23)
Paraguay	507 (424-611)	17.7 (14.93-20.79)	1189 (991-1408)	18.75 (15.78-21.96)
Peru	7464 (6213-8988)	46.16 (38.94-54.63)	14066 (11726-16648)	41.55 (34.83-48.79)
Philippines	9350 (7556-11468)	20.48 (16.93-24.53)	19310 (15600-23699)	19.04 (15.65-23.09)
Poland	21054 (17595-24756)	50.47 (42.4-59.16)	21951 (19660-24571)	40.86 (36.55-45.73)
Portugal	1805 (1522-2097)	14.96 (12.58-17.42)	2720 (2360-3121)	15.58 (13.35-17.99)
Puerto Rico	1192 (1003-1414)	33.04 (27.8-39.19)	1489 (1256-1738)	30.35 (25.31-36.14)
Qatar	100 (78-125)	29.92 (25.03-35.36)	734 (570-928)	28.69 (23.99-33.97)
Republic of Korea	10533 (8720-12721)	26.46 (22.27-31.18)	16904 (14039-19885)	24.02 (20.14-28.61)
Republic of Moldova	3397 (2895-3961)	75.91 (64.88-88.23)	3465 (2943-4025)	71.3 (60.85-82.73)
Romania	12245 (10293-14296)	48.05 (40.55-56.11)	14037 (12346-15927)	50.79 (44.66-57.15)

Russian Federation	122545 (104007-142485)	72.61 (62.04-84.58)	157599 (133616-183371)	81.97 (70.13-95.07)
Rwanda	977 (801-1203)	21.73 (18.3-25.73)	1966 (1590-2414)	21.4 (17.84-25.35)
Saint Kitts and Nevis	11 (9-13)	30.67 (25.88-36.16)	20 (17-24)	29.95 (25.21-35.1)
Saint Lucia	28 (23-34)	25.82 (21.29-30.8)	50 (41-60)	24.74 (20.33-29.84)
Saint Vincent and the Grenadines	25 (21-31)	29.38 (24.54-35.08)	36 (30-42)	28.58 (23.81-33.96)
Samoa	30 (25-37)	26.78 (22.37-31.79)	45 (38-54)	26.41 (22.04-31.47)
San Marino	6 (5-7)	21.36 (17.84-25.34)	10 (8-11)	20.69 (17.35-24.48)
Sao Tome and Principe	20 (17-24)	24.71 (21.1-29.05)	38 (31-45)	23.73 (20.04-28.02)
Saudi Arabia	2891 (2361-3551)	28.83 (24.08-34.25)	8606 (6886-10575)	27.59 (23.21-32.56)
Senegal	1205 (1000-1463)	25.1 (21.27-29.57)	2757 (2324-3315)	25.49 (21.87-29.81)
Serbia	4282 (3596-5062)	40.37 (34.01-47.34)	4388 (3872-4969)	35.49 (31.29-40.34)
Seychelles	15 (13-19)	24.39 (20.15-29.18)	27 (22-33)	23.84 (19.82-28.39)
Sierra Leone	594 (493-719)	23.11 (19.5-27.3)	1459 (1210-1755)	25.38 (21.56-29.65)
Singapore	317 (302-332)	11.86 (11.35-12.45)	1304 (1248-1362)	17.19 (16.4-18)
Slovakia	4415 (3961-4869)	78.08 (70.01-86.05)	4950 (4476-5467)	68.37 (61.86-75.22)
Slovenia	1075 (951-1219)	47.09 (41.72-53.38)	1237 (1119-1361)	38.84 (35.26-42.72)
Solomon Islands	50 (40-61)	24.59 (20.37-29.3)	110 (89-134)	24.03 (19.91-28.54)
Somalia	863 (694-1065)	19.46 (16.28-23.16)	2413 (1930-2981)	19.56 (16.31-23.27)
South Africa	6594 (5399-8062)	22.9 (19.18-27.4)	11715 (9672-14190)	22.19 (18.45-26.56)
South Sudan	754 (610-934)	19.95 (16.61-23.76)	1211 (981-1496)	19.8 (16.35-23.81)
Spain	15592 (14543-16821)	32.75 (30.32-35.7)	17925 (15419-20573)	25.26 (21.51-29.27)
Sri Lanka	3003 (2423-3686)	20.53 (16.93-24.78)	4694 (3835-5656)	19.43 (15.87-23.34)
Sudan	3328 (2736-4053)	26.16 (21.76-30.86)	7298 (5989-8892)	26.46 (22.16-31.2)
Suriname	95 (78-115)	29.52 (24.44-35.15)	177 (147-210)	29.56 (24.7-35.22)
Sweden	4147 (3480-4890)	37.27 (31.18-44.16)	6187 (5183-7286)	43.7 (36.63-51.74)
Switzerland	2139 (1938-2358)	24.34 (21.96-26.93)	3201 (2895-3540)	24.98 (22.55-27.6)

Syrian Arab Republic	2130 (1732-2606)	27.64 (22.89-32.63)	3542 (2904-4271)	26.35 (21.95-31.21)
Taiwan (Province of China)	9447 (8173-10860)	51.66 (44.71-58.93)	20027 (17863-22442)	58.65 (52.55-65.37)
Tajikistan	1032 (854-1231)	29.22 (24-34.61)	2148 (1737-2590)	29.29 (24.09-34.64)
Thailand	11375 (9284-13813)	23.27 (19.29-27.91)	20654 (16880-24536)	22.92 (18.91-27.39)
Timor-Leste	130 (105-160)	24.81 (20.57-29.85)	248 (204-301)	24.71 (20.4-29.38)
Togo	527 (436-649)	24.05 (20.44-28.31)	1451 (1214-1742)	25.19 (21.6-29.29)
Tokelau	0 (0-0)	26.09 (21.68-31.18)	0 (0-0)	25.98 (21.52-30.99)
Tonga	19 (16-23)	28.23 (23.6-33.34)	24 (20-29)	28.03 (23.35-33.25)
Trinidad and Tobago	290 (237-354)	27.91 (22.98-33.46)	447 (370-531)	27.29 (22.64-32.69)
Tunisia	1735 (1436-2102)	27.72 (23.11-32.89)	3449 (2820-4120)	27.32 (22.73-32.59)
Turkey	11822 (9739-14265)	26.22 (21.86-30.89)	23399 (20423-26551)	26.03 (22.84-29.57)
Turkmenistan	750 (623-904)	29.91 (24.74-35.54)	1475 (1201-1765)	31.37 (25.89-37.25)
Tuvalu	2 (2-2)	25.61 (21.31-30.48)	3 (2-3)	25.95 (21.6-30.77)
Uganda	2088 (1691-2593)	20.17 (16.82-23.94)	5035 (4076-6180)	20.1 (16.79-23.82)
Ukraine	43372 (36594-50468)	69.9 (59.19-81.52)	46531 (39205-54465)	77.04 (65.48-90.03)
United Arab Emirates	371 (286-467)	28.06 (23.5-33.25)	2636 (1969-3389)	27.51 (22.91-32.64)
United Kingdom	21086 (18423-24013)	29.44 (25.77-33.59)	31122 (27588-35057)	34.6 (30.52-39.03)
United Republic of Tanzania	3246 (2627-4007)	19.79 (16.45-23.56)	7699 (6253-9450)	20.06 (16.58-23.89)
United States of America	183032 (158101-211380)	62.9 (54.28-72.38)	228699 (210600-250346)	51.53 (47.37-56.19)
United States Virgin Islands	28 (23-34)	27.9 (23.06-33.26)	37 (31-43)	27.46 (22.74-32.97)
Uruguay	1089 (912-1281)	31.4 (26.19-37.15)	1281 (1075-1503)	29.6 (24.8-34.77)
Uzbekistan	4312 (3574-5167)	30 (24.87-35.51)	8818 (7168-10662)	30.39 (25.12-35.99)
Vanuatu	29 (24-35)	29.38 (24.55-34.54)	65 (54-78)	29.25 (24.6-34.39)
Venezuela (Bolivarian Republic of)	5491 (4497-6670)	37.33 (31.03-44.73)	10494 (8612-12580)	35.58 (29.3-42.69)
Viet Nam	15445 (12752-18743)	29.97 (24.82-35.69)	31786 (26925-37163)	30.25 (26-35.01)

Yemen	2021 (1655-2505)	26.79 (22.43-31.66)	5560 (4548-6833)	27.24 (22.75-32.33)
Zambia	992 (807-1218)	20.83 (17.37-24.75)	2359 (1894-2913)	19.78 (16.5-23.55)
Zimbabwe	1251 (1010-1560)	19.33 (16.09-23.12)	2145 (1746-2645)	19.99 (16.69-23.91)

ASIR: age-standardized incident rate

Table S5 The deaths and ASDR of pancreatitis in 1990 and 2019 among all countries/territories.

Country/region	Deaths in 1990	ASDR in 1990	Deaths in 2019	ASDR in 2019
Afghanistan	114 (72-190)	1.81 (1.12-3.22)	185 (125-287)	1.55 (0.96-2.63)
Albania	22 (18-35)	1.04 (0.87-1.58)	28 (20-42)	0.72 (0.5-1.07)
Algeria	100 (71-146)	1.03 (0.73-1.48)	208 (135-277)	0.75 (0.48-1)
American Samoa	0 (0-1)	1.52 (1.12-2.3)	1 (1-1)	1.6 (1.21-2.03)
Andorra	1 (0-1)	1.11 (0.76-1.67)	1 (1-2)	0.94 (0.6-1.48)
Angola	81 (52-140)	1.75 (1.1-3.06)	197 (118-326)	1.5 (0.91-2.54)
Antigua and Barbuda	1 (0-1)	0.98 (0.86-1.14)	1 (1-1)	0.91 (0.75-1.09)
Argentina	962 (844-1064)	3.03 (2.65-3.34)	1042 (917-1261)	1.97 (1.73-2.37)
Armenia	17 (15-20)	0.64 (0.56-0.73)	31 (25-38)	0.81 (0.66-0.98)
Australia	179 (165-196)	0.94 (0.86-1.03)	280 (239-339)	0.65 (0.56-0.78)
Austria	195 (165-210)	1.76 (1.46-1.9)	126 (108-171)	0.73 (0.64-0.95)
Azerbaijan	40 (29-51)	0.74 (0.55-0.96)	65 (48-87)	0.7 (0.51-1.01)
Bahamas	4 (3-5)	2.27 (1.94-2.68)	8 (6-10)	2.02 (1.61-2.56)
Bahrain	3 (2-4)	2.09 (1.62-2.56)	8 (5-11)	1.36 (0.88-1.74)
Bangladesh	1747 (1272-2360)	3.19 (2.31-4.34)	2459 (1382-3753)	1.83 (1.07-2.77)
Barbados	3 (3-4)	1.19 (1.03-1.37)	5 (4-7)	1.17 (0.91-1.46)
Belarus	368 (318-421)	3.01 (2.6-3.44)	586 (432-783)	4.28 (3.12-5.75)
Belgium	222 (185-242)	1.51 (1.25-1.63)	203 (173-251)	0.88 (0.76-1.07)
Belize	1 (1-1)	0.86 (0.59-1.14)	3 (3-4)	1.02 (0.86-1.21)
Benin	69 (49-100)	3.08 (2.19-4.48)	203 (144-289)	3.33 (2.45-4.59)
Bermuda	1 (1-1)	1.79 (1.48-2.14)	1 (1-1)	0.92 (0.73-1.15)
Bhutan	7 (4-11)	2.32 (1.47-3.63)	11 (6-20)	1.96 (1.13-3.42)
Bolivia (Plurinational State of)	212 (143-301)	5.89 (4.09-8.52)	356 (255-498)	4.08 (2.94-5.71)
Bosnia and Herzegovina	90 (56-116)	2.32 (1.38-3.09)	121 (56-173)	2.12 (1.02-3.01)

Botswana	10 (6-16)	1.56 (0.95-2.45)	22 (14-32)	1.34 (0.89-1.9)
Brazil	2290 (2176-2455)	2.28 (2.15-2.47)	5445 (4725-5901)	2.3 (1.99-2.5)
Brunei Darussalam	2 (1-2)	1.39 (1.07-1.67)	3 (2-4)	1.12 (0.89-1.36)
Bulgaria	186 (163-249)	1.66 (1.47-2.25)	275 (213-344)	2.15 (1.66-2.73)
Burkina Faso	169 (122-227)	3.68 (2.7-4.9)	509 (297-850)	4.62 (2.72-7.64)
Burundi	48 (27-81)	1.83 (1.03-3.03)	97 (46-184)	1.81 (0.86-3.46)
Cabo Verde	5 (3-6)	2.17 (1.56-2.87)	12 (9-16)	2.54 (1.95-3.29)
Cambodia	99 (71-154)	1.87 (1.35-2.88)	178 (120-299)	1.46 (1.03-2.35)
Cameroon	189 (132-256)	3.71 (2.64-5.01)	525 (291-817)	3.48 (1.99-5.35)
Canada	298 (269-321)	0.94 (0.85-1.02)	546 (463-636)	0.79 (0.68-0.91)
Central African Republic	35 (20-74)	2.57 (1.49-5.13)	60 (28-134)	2.26 (1.14-4.62)
Chad	108 (69-157)	3.5 (2.22-5.11)	262 (171-381)	3.76 (2.5-5.42)
Chile	243 (222-265)	2.36 (2.15-2.57)	388 (343-437)	1.65 (1.46-1.85)
China	8976 (7191-12084)	1.08 (0.87-1.46)	10664 (8196-12810)	0.59 (0.46-0.7)
Colombia	275 (254-296)	1.43 (1.31-1.53)	531 (400-688)	1 (0.75-1.3)
Comoros	4 (2-9)	1.86 (0.82-3.69)	8 (4-15)	1.47 (0.81-2.99)
Congo	24 (16-40)	2 (1.33-3.27)	41 (23-69)	1.38 (0.79-2.34)
Cook Islands	0 (0-1)	2.58 (1.9-3.72)	0 (0-1)	1.73 (1.2-2.38)
Costa Rica	44 (33-50)	2.39 (1.74-2.69)	105 (78-135)	2.03 (1.52-2.61)
Croatia	158 (137-173)	2.63 (2.28-2.89)	143 (113-181)	1.72 (1.35-2.18)
Cuba	98 (88-113)	0.93 (0.84-1.08)	210 (165-267)	1.19 (0.92-1.51)
Cyprus	12 (9-15)	1.86 (1.22-2.37)	20 (12-25)	1.17 (0.67-1.47)
Czechia	421 (389-486)	3.23 (2.99-3.7)	394 (317-494)	2.05 (1.65-2.55)
Côte d'Ivoire	169 (111-256)	3.19 (2.18-4.7)	443 (292-654)	3.14 (2.08-4.59)
Democratic People's Republic of Korea	172 (112-275)	1.1 (0.75-1.72)	283 (182-444)	0.92 (0.6-1.44)

Democratic Republic of the Congo	298 (188-516)	1.72 (1.06-3.16)	593 (330-1143)	1.37 (0.74-2.77)
Denmark	117 (105-136)	1.59 (1.37-1.82)	158 (133-179)	1.46 (1.18-1.65)
Djibouti	2 (1-4)	1.36 (0.71-2.48)	8 (4-14)	1.12 (0.58-2.1)
Dominica	1 (1-2)	1.8 (1.43-2.33)	1 (1-2)	1.5 (1.14-2)
Dominican Republic	39 (30-48)	0.88 (0.68-1.11)	80 (53-112)	0.84 (0.57-1.16)
Ecuador	340 (222-441)	5.72 (3.66-7.44)	449 (326-699)	3.01 (2.19-4.7)
Egypt	299 (212-437)	1.21 (0.86-1.83)	571 (307-877)	1.1 (0.61-1.71)
El Salvador	85 (62-107)	2.57 (1.81-3.3)	93 (68-127)	1.53 (1.1-2.08)
Equatorial Guinea	5 (2-10)	2.17 (1.1-4.35)	7 (3-14)	1.26 (0.61-2.36)
Eritrea	17 (10-30)	1.44 (0.76-2.5)	51 (27-87)	1.58 (0.83-2.72)
Estonia	53 (43-60)	2.77 (2.24-3.12)	44 (32-58)	2.01 (1.49-2.61)
Eswatini	4 (3-7)	1.23 (0.87-1.96)	9 (5-14)	1.38 (0.81-2.04)
Ethiopia	454 (288-787)	2.05 (1.26-3.45)	625 (344-1232)	1.36 (0.74-2.75)
Fiji	4 (3-5)	0.85 (0.6-1.13)	4 (3-6)	0.61 (0.45-0.81)
Finland	137 (118-155)	2.08 (1.78-2.36)	179 (155-202)	1.8 (1.44-2.04)
France	1116 (1018-1205)	1.38 (1.27-1.48)	1186 (1024-1378)	0.85 (0.75-0.99)
Gabon	10 (7-15)	1.65 (1.15-2.52)	14 (9-23)	1.21 (0.79-2.01)
Gambia	16 (10-25)	3.78 (2.29-5.7)	37 (26-53)	3.23 (2.31-4.55)
Georgia	23 (19-27)	0.41 (0.35-0.48)	43 (29-54)	0.76 (0.51-0.94)
Germany	1994 (1767-2327)	1.67 (1.48-1.94)	2173 (1890-2785)	1.2 (1.05-1.49)
Ghana	299 (173-453)	4.14 (2.46-6.37)	754 (453-1109)	3.95 (2.38-5.7)
Greece	203 (172-223)	1.41 (1.19-1.55)	349 (270-407)	1.28 (1.06-1.45)
Greenland	1 (1-1)	2.11 (1.35-2.66)	1 (1-1)	1.33 (1.02-1.74)
Grenada	1 (1-1)	1 (0.85-1.18)	1 (1-1)	0.9 (0.77-1.07)
Guam	1 (0-1)	0.77 (0.61-0.96)	1 (1-1)	0.59 (0.39-0.77)

Guatemala	230 (182-275)	4.76 (3.89-5.73)	396 (304-512)	2.96 (2.27-3.85)
Guinea	100 (69-149)	2.81 (1.93-4.31)	183 (122-265)	2.84 (1.92-4.15)
Guinea-Bissau	25 (14-40)	5.17 (3.09-8.01)	45 (29-65)	4.78 (3.15-6.79)
Guyana	11 (9-13)	2.37 (1.98-2.91)	16 (12-20)	2.37 (1.78-3)
Haiti	95 (66-141)	2.61 (1.7-4.1)	157 (102-242)	1.98 (1.28-3.08)
Honduras	97 (68-128)	3.72 (2.58-5.33)	253 (179-353)	4.09 (2.91-5.55)
Hungary	568 (520-602)	4.29 (3.87-4.57)	379 (307-471)	2.18 (1.75-2.72)
Iceland	3 (3-4)	1.13 (1-1.26)	4 (3-4)	0.66 (0.56-0.78)
India	10536 (8508-15282)	2.09 (1.68-3)	20456 (15270-25750)	1.75 (1.33-2.19)
Indonesia	2278 (1672-3614)	2.16 (1.59-3.36)	3440 (2629-5523)	1.77 (1.38-2.68)
Iran (Islamic Republic of)	171 (125-258)	0.83 (0.57-1.28)	398 (308-480)	0.6 (0.45-0.71)
Iraq	42 (29-72)	0.51 (0.35-0.91)	99 (73-136)	0.43 (0.32-0.65)
Ireland	30 (28-36)	0.77 (0.71-0.9)	48 (41-55)	0.66 (0.56-0.75)
Israel	45 (37-51)	0.99 (0.8-1.11)	109 (87-128)	0.89 (0.73-1.03)
Italy	984 (917-1209)	1.16 (1.08-1.44)	1305 (1137-1481)	0.82 (0.73-0.97)
Jamaica	11 (9-13)	0.6 (0.5-0.7)	18 (13-22)	0.58 (0.44-0.73)
Japan	1272 (1180-1356)	0.81 (0.75-0.86)	1655 (1318-2232)	0.44 (0.38-0.57)
Jordan	20 (15-25)	1.75 (1.3-2.24)	47 (37-59)	0.86 (0.67-1.1)
Kazakhstan	830 (685-928)	6.33 (5.07-7.13)	881 (679-1074)	4.97 (3.75-6.01)
Kenya	155 (70-265)	1.74 (0.78-3.07)	432 (232-702)	1.79 (0.94-2.95)
Kiribati	2 (1-2)	3.73 (2.63-6.14)	2 (2-3)	2.93 (2.03-4.53)
Kuwait	7 (6-8)	0.96 (0.81-1.11)	22 (18-28)	0.82 (0.65-1.03)
Kyrgyzstan	41 (36-52)	1.26 (1.11-1.64)	86 (68-103)	1.57 (1.28-1.87)
Lao People's Democratic Republic	44 (28-69)	1.87 (1.26-2.8)	68 (43-105)	1.39 (0.91-2.1)
Latvia	101 (91-114)	3.08 (2.78-3.46)	100 (81-126)	3.33 (2.64-4.2)

Lebanon	18 (12-25)	0.94 (0.62-1.32)	36 (21-52)	0.71 (0.41-1.02)
Lesotho	14 (9-22)	1.36 (0.88-2.07)	23 (15-34)	1.66 (1.14-2.32)
Liberia	37 (23-56)	3.24 (2.05-4.84)	84 (52-123)	3.15 (2.07-4.43)
Libya	19 (11-29)	1.06 (0.56-1.7)	39 (25-56)	0.82 (0.49-1.17)
Lithuania	106 (93-122)	2.48 (2.18-2.87)	184 (132-231)	3.97 (2.88-5.08)
Luxembourg	7 (6-8)	1.3 (1.18-1.44)	8 (6-10)	0.77 (0.64-0.96)
Madagascar	63 (38-103)	1.09 (0.62-1.91)	148 (84-264)	1.17 (0.66-2.17)
Malawi	66 (44-104)	1.56 (1-2.52)	130 (75-237)	1.54 (0.89-2.81)
Malaysia	238 (198-298)	2.47 (2-3.01)	635 (469-838)	2.49 (1.81-3.25)
Maldives	1 (1-1)	1.03 (0.65-1.43)	2 (1-2)	0.47 (0.34-0.59)
Mali	144 (95-230)	3.1 (2.06-4.85)	356 (229-567)	3.47 (2.29-5.45)
Malta	5 (4-5)	1.12 (0.99-1.23)	7 (6-8)	0.76 (0.65-0.9)
Marshall Islands	0 (0-1)	2.28 (1.57-3.53)	1 (0-1)	1.73 (1.11-2.76)
Mauritania	44 (31-61)	4.02 (2.87-5.48)	56 (37-85)	2.47 (1.71-3.6)
Mauritius	31 (27-35)	3.33 (2.93-3.71)	31 (23-41)	1.92 (1.45-2.5)
Mexico	1103 (1055-1195)	2.21 (2.1-2.48)	2511 (2126-2977)	2.11 (1.79-2.5)
Micronesia (Federated States of)	1 (1-2)	2.4 (1.69-3.44)	1 (1-2)	1.79 (1.11-2.85)
Monaco	1 (0-1)	0.72 (0.51-0.99)	1 (0-1)	0.65 (0.46-0.86)
Mongolia	60 (27-92)	5.76 (2.39-9.14)	105 (55-149)	4.47 (2.1-6.33)
Montenegro	10 (9-13)	1.65 (1.36-2.12)	14 (11-17)	1.54 (1.24-1.95)
Morocco	130 (83-199)	1.1 (0.62-1.81)	251 (172-344)	0.96 (0.62-1.32)
Mozambique	103 (63-176)	1.57 (0.95-2.87)	274 (157-515)	2.07 (1.2-3.9)
Myanmar	316 (192-582)	1.11 (0.71-1.92)	364 (232-673)	0.72 (0.47-1.3)
Namibia	11 (7-16)	1.49 (0.94-2.16)	21 (13-31)	1.33 (0.89-1.92)
Nauru	0 (0-0)	2.16 (1.32-3.48)	0 (0-0)	1.8 (1.09-2.85)
Nepal	354 (246-506)	3.41 (2.26-5.04)	531 (347-739)	2.48 (1.63-3.47)

Netherlands	188 (173-215)	0.96 (0.88-1.09)	269 (235-315)	0.79 (0.7-0.92)
New Zealand	36 (31-40)	0.95 (0.82-1.03)	59 (49-68)	0.74 (0.62-0.84)
Nicaragua	28 (21-33)	1.6 (1.16-1.93)	68 (47-89)	1.55 (1.09-2)
Niger	126 (85-193)	3.51 (2.33-5.27)	361 (216-572)	3.83 (2.32-6.01)
Nigeria	2060 (1113-3949)	4.11 (2.23-7.87)	3882 (2087-7494)	3.67 (2.06-6.91)
Niue	0 (0-0)	1.58 (1.13-2.26)	0 (0-0)	1.27 (0.89-1.74)
North Macedonia	30 (25-40)	1.64 (1.35-2.19)	49 (36-64)	1.7 (1.25-2.19)
Northern Mariana Islands	0 (0-1)	1.03 (0.65-2)	1 (0-1)	1.27 (0.84-1.63)
Norway	66 (53-72)	0.97 (0.78-1.05)	70 (57-80)	0.7 (0.58-0.79)
Oman	4 (2-6)	0.63 (0.32-1.07)	6 (3-8)	0.44 (0.25-0.59)
Pakistan	1406 (910-2037)	2.33 (1.47-3.46)	2479 (1542-3465)	2.09 (1.31-2.89)
Palau	0 (0-0)	1.38 (0.83-2.19)	0 (0-0)	1.28 (0.77-1.92)
Palestine	13 (9-18)	1.65 (1.18-2.29)	23 (17-29)	1.27 (0.88-1.61)
Panama	15 (12-17)	0.92 (0.75-1.04)	35 (26-46)	0.84 (0.61-1.11)
Papua New Guinea	20 (11-30)	0.86 (0.5-1.36)	50 (30-75)	0.84 (0.51-1.29)
Paraguay	40 (25-51)	1.71 (1.03-2.2)	112 (54-163)	2.01 (0.92-2.93)
Peru	525 (393-656)	3.68 (2.84-4.67)	640 (451-870)	1.94 (1.37-2.66)
Philippines	397 (318-507)	1.11 (0.82-1.38)	766 (587-1022)	0.91 (0.7-1.16)
Poland	1416 (1338-1799)	3.35 (3.16-4.27)	2003 (1666-2363)	3.23 (2.68-3.83)
Portugal	244 (224-276)	1.92 (1.76-2.15)	354 (290-402)	1.39 (1.17-1.57)
Puerto Rico	88 (70-98)	2.52 (1.96-2.81)	102 (77-132)	1.48 (1.11-1.91)
Qatar	2 (1-3)	1.72 (1.1-2.73)	10 (5-15)	1.3 (0.79-1.8)
Republic of Korea	503 (348-734)	1.73 (1.2-2.29)	562 (415-694)	0.69 (0.51-0.84)
Republic of Moldova	358 (322-393)	7.81 (7.09-8.57)	230 (191-290)	4.43 (3.66-5.51)
Romania	968 (869-1163)	3.67 (3.29-4.41)	1043 (837-1309)	3.31 (2.66-4.11)
Russian Federation	4786 (4254-7789)	2.77 (2.45-4.48)	11615 (9804-13619)	5.7 (4.79-6.72)

Rwanda	77 (49-119)	2.4 (1.47-3.76)	103 (58-173)	1.52 (0.88-2.56)
Saint Kitts and Nevis	1 (1-2)	3.57 (2.89-4.49)	2 (1-2)	2.66 (1.94-3.46)
Saint Lucia	1 (0-1)	0.62 (0.52-0.71)	1 (1-1)	0.5 (0.4-0.6)
Saint Vincent and the Grenadines	1 (1-1)	1.5 (1.3-1.72)	2 (2-2)	1.44 (1.17-1.72)
Samoa	1 (1-2)	1.58 (1.12-2.35)	2 (1-3)	1.33 (0.98-1.8)
San Marino	0 (0-1)	1.29 (0.97-1.67)	1 (1-1)	1.11 (0.71-1.62)
Sao Tome and Principe	2 (1-3)	2.83 (1.77-4.75)	3 (2-5)	2.35 (1.53-3.89)
Saudi Arabia	101 (59-174)	1.97 (1.12-3.43)	166 (115-251)	1.12 (0.83-1.65)
Senegal	118 (80-182)	3.26 (2.27-4.93)	277 (176-426)	3.21 (2.09-4.97)
Serbia	311 (252-378)	2.88 (2.37-3.48)	399 (258-520)	2.69 (1.81-3.49)
Seychelles	1 (1-1)	1.39 (0.91-2.13)	1 (1-2)	1.28 (0.75-2.18)
Sierra Leone	55 (34-90)	2.66 (1.61-4.32)	150 (98-229)	3.4 (2.27-5.2)
Singapore	16 (14-17)	0.73 (0.62-0.8)	28 (23-32)	0.38 (0.31-0.43)
Slovakia	230 (197-309)	4 (3.43-5.29)	237 (177-313)	2.81 (2.09-3.72)
Slovenia	66 (48-85)	2.82 (2.06-3.61)	56 (43-73)	1.29 (0.98-1.69)
Solomon Islands	2 (1-4)	1.37 (0.85-2.09)	5 (3-7)	1.21 (0.85-1.73)
Somalia	43 (25-77)	1.43 (0.84-2.52)	98 (55-182)	1.21 (0.66-2.36)
South Africa	240 (191-303)	0.96 (0.77-1.22)	389 (314-469)	0.81 (0.66-0.97)
South Sudan	35 (19-64)	1.35 (0.72-2.58)	47 (24-91)	1.15 (0.58-2.24)
Spain	1161 (1057-1246)	2.24 (2.04-2.4)	1592 (1359-1820)	1.47 (1.29-1.68)
Sri Lanka	93 (69-123)	0.76 (0.56-0.97)	97 (67-136)	0.41 (0.29-0.56)
Sudan	80 (43-133)	0.94 (0.46-1.67)	166 (83-263)	0.97 (0.45-1.56)
Suriname	5 (4-6)	1.72 (1.45-2.22)	10 (7-13)	1.65 (1.24-2.1)
Sweden	130 (117-141)	0.94 (0.84-1.01)	184 (138-208)	0.87 (0.65-0.97)
Switzerland	70 (58-81)	0.68 (0.56-0.78)	106 (89-128)	0.58 (0.49-0.68)
Syrian Arab Republic	47 (22-69)	1 (0.41-1.54)	70 (32-105)	0.7 (0.33-1.03)

Taiwan (Province of China)	177 (163-202)	1.1 (1.01-1.27)	343 (263-440)	0.94 (0.72-1.2)
Tajikistan	20 (13-30)	0.7 (0.41-1.1)	35 (25-46)	0.89 (0.48-1.28)
Thailand	685 (503-970)	1.66 (1.24-2.28)	1254 (858-1720)	1.33 (0.91-1.82)
Timor-Leste	6 (3-10)	1.44 (0.77-2.55)	11 (6-17)	1.3 (0.76-2.09)
Togo	45 (31-63)	2.96 (2.07-4.04)	171 (104-265)	3.65 (2.36-5.52)
Tokelau	0 (0-0)	1.43 (0.99-2.28)	0 (0-0)	1.15 (0.84-1.58)
Tonga	1 (1-1)	1.79 (1.21-2.69)	1 (1-2)	1.57 (1.16-2.38)
Trinidad and Tobago	9 (8-11)	1.04 (0.92-1.17)	17 (12-23)	0.95 (0.69-1.29)
Tunisia	33 (21-51)	0.77 (0.49-1.2)	77 (48-108)	0.68 (0.42-0.96)
Turkey	456 (359-638)	1.35 (1.04-1.91)	832 (588-1048)	1 (0.71-1.26)
Turkmenistan	21 (17-28)	0.98 (0.81-1.31)	63 (49-80)	1.42 (1.11-1.79)
Tuvalu	0 (0-0)	1.85 (1.26-2.98)	0 (0-0)	1.49 (1.04-2.18)
Uganda	112 (59-185)	1.59 (0.83-2.7)	245 (134-418)	1.47 (0.82-2.51)
Ukraine	1889 (1679-2135)	2.91 (2.59-3.29)	2819 (2260-3445)	4.65 (3.7-5.71)
United Arab Emirates	6 (3-9)	1.21 (0.6-1.83)	40 (19-71)	0.87 (0.39-1.44)
United Kingdom	1046 (941-1276)	1.21 (1.1-1.48)	1524 (1385-1802)	1.25 (1.15-1.44)
United Republic of Tanzania	160 (98-249)	1.34 (0.81-2.16)	383 (216-752)	1.36 (0.75-2.73)
United States of America	3049 (2847-3273)	0.98 (0.91-1.05)	4897 (4495-5385)	0.93 (0.87-1.02)
United States Virgin Islands	1 (1-1)	1.15 (0.92-1.6)	2 (1-2)	1.09 (0.8-1.39)
Uruguay	62 (55-68)	1.66 (1.49-1.83)	66 (58-78)	1.31 (1.16-1.53)
Uzbekistan	128 (101-165)	0.99 (0.78-1.33)	345 (277-430)	1.4 (1.14-1.73)
Vanuatu	1 (1-2)	1.93 (1.21-3.03)	3 (2-5)	1.78 (1.24-2.64)
Venezuela (Bolivarian Republic of)	141 (126-160)	1.26 (1.11-1.45)	340 (247-445)	1.18 (0.86-1.54)
Viet Nam	798 (508-1275)	1.94 (1.23-3.07)	1056 (678-1818)	1.16 (0.76-1.95)
Yemen	54 (28-90)	1.3 (0.59-2.28)	137 (79-217)	1.15 (0.6-1.88)

Zambia	58 (38-88)	1.82 (1.14-2.75)	118 (78-182)	1.46 (0.96-2.24)
Zimbabwe	46 (30-62)	1.07 (0.68-1.41)	111 (65-163)	1.38 (0.83-1.99)

ASDR: age-standardized death rate

Table S6 Net drift for age-period-cohort model of global pancreatitis incidence and mortality

	Sex	Net drift	95% CI	Wald tests P-value
Incidence	Both	-0.321	-0.35 to -0.291	P < 0.001
	Male	-0.206	-0.239 to -0.173	P < 0.001
	Female	-0.426	-0.458 to -0.394	P < 0.001
Deaths	Both	-0.769	-0.811 to -0.727	P < 0.001
	Male	-0.674	-0.734 to -0.615	P < 0.001
	Female	-1.047	-1.117 to -0.977	P < 0.001

CI: confidence interval
